# Acetylcholinesterase Inhibitory Meroterpenoid from a Mangrove Endophytic Fungus *Aspergillus* sp. 16-5c

**DOI:** 10.3390/molecules22050727

**Published:** 2017-05-03

**Authors:** Yuhua Long, Hui Cui, Xinglie Liu, Ze’en Xiao, Shitong Wen, Zhigang She, Xishan Huang

**Affiliations:** 1 School of Chemistry, Sun Yat-Sen University, Guangzhou 510275, China; yuhualong68@hotmail.com (Y.L.); cuihui2@mail2.sysu.edu.cn (H.C.); xiaozeen@mail2.sysu.edu.cn (Z.X.); cesshzhg@mail.sysu.edu.cn (Z.S.); 2 School of Chemistry and Environment, South China Normal University, Guangzhou 510006, China; wenshitong123@163.com; 3 The Sixth Affiliated Hospital of Sun Yat-sen University, Guangzhou 510275, China; 13922342800@163.com

**Keywords:** meroterpenoids, *Aspergillus* sp., acetylcholinesterase (AchE) inhibitory

## Abstract

One new meroterpenoid, named 2-hydroacetoxydehydroaustin (**1**), together with nine known meroterpenoids, 11-acetoxyisoaustinone (**2**), isoaustinol (**3**), austin (**4**), austinol (**5**), acetoxydehydroaustin (**6**), dehydroaustin (**7**), dehydroaustinol (**8**), preaustinoid A2 (**9**), and 1,2-dihydro-acetoxydehydroaustin B (**10**), were isolated from the mangrove endophytic fungus, *Aspergillus* sp. 16-5c. These structures were characterized by spectroscopic analysis, further the absolute configurations of stereogenic carbons for Compounds **1**, **3**, **4**, **6**, **7**, **8**, **9**, and **10** were determined by single crystal X-ray diffraction analysis using Cu Kα radiation. Moreover, the absolute configurations of stereogenic carbons for Known Compounds **3**, **7**, **8**, and **9** are identified here for the first time. Compounds **3**, **7**, and **8** showed acetylcholinesterase (AchE) inhibitory activity with IC_50_ values of 2.50, 0.40, and 3.00 μM, respectively.

## 1. Introduction

**Figure 1:**
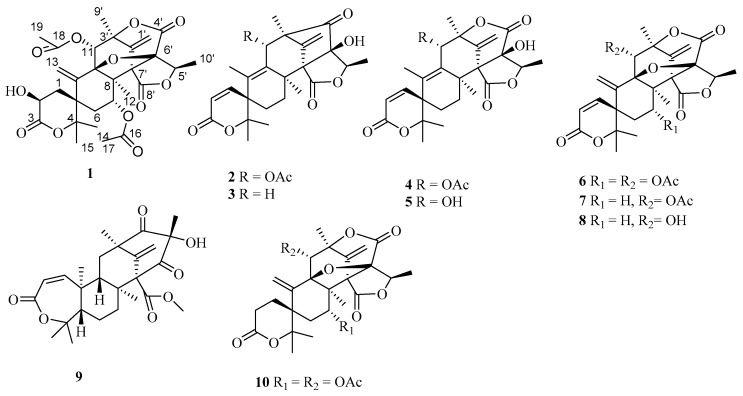
Structures of Compounds **1**–**10**.

**Figure 2:**
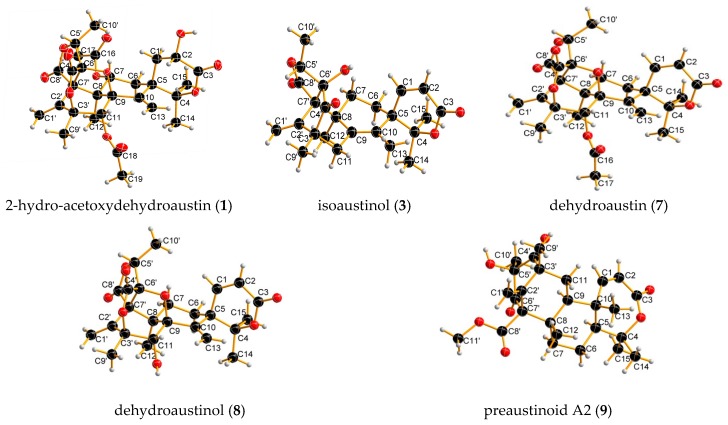
Perspective ORTEP drawings for **1**, **3**, **7**, **8**, and **9**.

Meroterpenoids are natural products derived from terpenoids mixed biosynthetic origin [1]. Meroterpenoids display structural diversity with widespread biological activities including antimicrobial, antiviral, and antitumoral activities and enzyme inhibition. Despite the structural diversity, austin and austin analogues are mixed polyketide–terpenoid meroterpenoids derived from 3,5-dimethylorsellinic acid, which were most often isolated from fungi [2]. Austin was first isolated as a novel polyisoprenoid mycotoxin from *Aspergillus ustus* in 1976 [3]. Subsequently, some austin analogues from *Aspergillus* sp. and *Penicillium* sp. have been reported [2,3,4,5,6,7,8,9,10,11,12,13,14,15,16]. As part of our ongoing investigations on novel bioactive compounds from mangrove endophytic-derived fungi [17,18,19,20], ten meroterpenoids, including one new meroterpenoids named 2-hydro-acetoxydehydroaustin (**1**), together with nine known meroterpenoids, 11-acetoxyisoaustinone (**2**), isoaustinol (**3**), austin (**4**), austinol (**5**), acetoxydehydroaustin (**6**), dehydroaustin (**7**), dehydroaustinol (**8**), preaustinoid A2 (**9**), and 1,2-dihydro-acetoxydehydroaustin B (**10**), were isolated from the fungus *Aspergillus* 16-5c, which was isolated from the leaves of the mangrove plant *Sonneratia apetala* collected on the coastal saltmarsh of the South China Sea (Figure 1). Compound **10** was reported as a mixture previously [16], but it is reported as a single compound here. The absolute configurations of stereogenic carbons for Compounds **3**, **7**, **8**, and **9** are determined here for the first time (Figure 2). Herein, we report the isolation, structural elucidation, acetylcholinesterase (AChE) inhibitory activity, and cytotoxicity of these meroterpenoids.

## 2. Results and Discussion

Compound **1** was obtained as colorless crystal, m.p. >300 °C. The HRESIMS result (*m/z* 575.2124 [M + H]^+^) suggested the molecular formula of Compound **1** as C_29_H_34_O_12_ with 13 degrees of unsaturation. The data of ^1^D-NMR (Table 1) combined with HSQC spectrum showed that **1** has seven methyl groups [δ_H_ 1.37, 1.43, 1.47, 1.58, 1.70, 2.05, 2.07 (each 3H, s)], two methylene groups, four oxygenated methines (δ_H_ 4.28/*δ*_C_ 64.8, δ_H_ 5.35/δ_C_ 67.8, δ_H_ 5.70/δ*_C_* 74.3, and δ_H_ 5.26/δ_C_ 76.6), one terminal olefin moiety [δ_H_ 5.81, 5.85 (each 1H, *J* = 1.5 Hz) and δ_H_ 5.73, 6.14 (each 1H, s)], two acetyl groups (δ_H_ 2.05/δ_C_ 20.8, 170.6 and δ_H_ 2.07/δ_C_ 21.1, 168.9), and three others.

**Figure 3:**
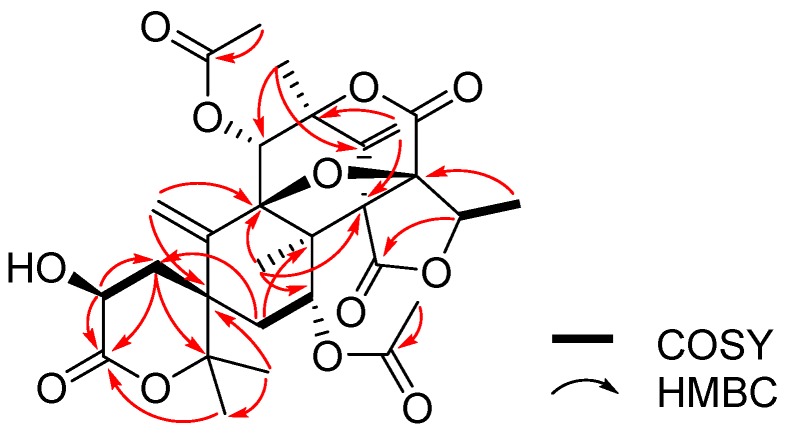
Selected ^1^H-^1^H COSY (bold line) and HMBC (arrow) correlations of Compound **1**.

The above spectroscopic features suggested that **1** belonged to the austin analogue [16], whose planar structure is similar to the 1,2-dihydro-acetoxydehydroaustin B. However, the carbon chemical shifts values of C-1 (δ_C_ 37.1) and C-2 (δ_C_ 64.8) were different from those of 1,2-dihydro-acetoxydehydroaustin B. The detailed comparison of the NMR data of **1** with those of 1,2-dihydro-acetoxydehydroaustin B clearly confirmed that **1** was a new structure (Figure 3). The absolute configurations of strereogenic carbons for **1** were determined by single-crystal X-ray diffraction using Cu Kα radiation [21]. Therefore, Compound **1** was named 2-hydroacetoxydehydroaustin. Its absolute configurations were 1S, 7R, 8S, 9R, 11S, 3′R, 5′R, 7′R.

Compound **2** was obtained as a colorless powder. Its molecular formula was assigned to be C_27_H_32_O_8_ from the HRESIMS molecular ion peak at *m/z* 507.1988 [M + Na]^+^. Comparison of the NMR data revealed that the structure of **2** resembled that of **3** except for the presence of an acetyl group. A carbon signal at δ_C_ 74.1 (C-11) was observed in **2** compared to that of **3** [7]. It was deduced that the acetyl group was connected to C-11 via the hydroxyl group in **2** supported by the HMBC correlation from H-11 (δ_H_ 5.74, 1H, s) to 11-CH_3_CO (δ_C_ 171.5). The relative configuration was confirmed by the NOE correlations, and the correlation signals between 9-CH_3_ (δ_H_ 1.25), 11-OCOCH_3_ (δ_H_ 2.04), and 12-CH_3_ (δ_H_ 1.57) showed that the 11-acetoxyl, 12 and 9’-CH_3_ were oriented at the same side. Furthermore, the NOE correlations between 10’-CH_3_ (δ_H_ 1.30) and 6’-OH (δ_H_ 2.88) supported that the methyl and hydroxyl groups were at the same side. The NOE correlations of **2** were the same as **3**, in addition to their positive optical rotation values. Finally, the absolute configurations of stereogenic carbons for **2** were assigned as 5R, 8S, 11S, 3′R, 5′R, 6′R, 7′R. These results confirmed that **2** was consistent with 11-acetoxylisoaustinone [22].

Austin was first isolated from *Aspergillus ustus* in 1976 before the Flack constant (the absolute configurations were determined) reported in 1983, and its absolute configuration was not definite. Herein, the absolute configurations of stereogenic carbons for Known Compounds **3**, **7**, **8**, and **9** are identified for the first time by single crystal X-ray diffraction analysis using Cu Kα radiation. The absolute configurations were shown as follows: **3** is 5R, 8S, 3′R, 5′R, 6′R, 7′R, **7** is 5R, 8R, 11S, 3′S, 5′R, 6′R, 7′R, **8** is 5R, 8R, 11S, 3′S, 5′R, 6′R, 7′R, and **9** is 5S, 8S, 9S, 10S, 3′R, 5′S, 7′R (see the Supplementary Materials).

Although meroterpenoids have a wide range of biological activities [2,3,4,5,6,7,8,9,10,11,12,13,14,15,16], few biological activities have been reported for austin and austin analogues. In this work, Compounds **1**–**10** were evaluated for their acetylcholinesterase (AChE) inhibitory activity using Huperzine A as a control (Table 2). The results showed Compounds **3**, **7**, and **8** were potential AChE inhibitors with IC_50_ values of 2.50, 0.40, and 3.00 μM, respectively.

In addition, four human cancer cell lines (human breast cancer cell lines MCF-7 and MDA-MB-435, human hepatoma cell line HepG2, and human prostatic cancer cell line PC-3) were used in the in vitro cytotoxicity bioassay. The results showed that Compounds **1**–**10** have no cytotoxicity (>50 μM) against human cancer cell lines.

## 3. Experimental Section

### 3.1. General

Melting points were determined on an X-4 micromelting point apparatus and are uncorrected. Optical rotations were measured on a Polartronic HHW5 digital polarimeter. IR spectra were measured on a Bruker Vector 22 spectrophotometer (Bruker, Billerica, MA, USA) using KBr pellets. The NMR spectra were recorded on a Bruker Avance 400 spectrometer at 400 MHz for ^1^H and 100 MHz for ^13^C in CDCl_3_. All chemical shifts (δ) are given in ppm with reference to the solvent signal (CDCl_3_, δ_H_ 7.26 for ^1^H, δ_C_ 77.23 for ^13^C; DMSO, δ_H_ 2.50 for ^1^H, δ_C_ 39.52 for ^13^C), and coupling constants (*J*) are given in Hz. HRESIMS spectra were recorded on a Finnigan LCQ-DECA mass spectrometer (Thermo Scientific, shanghai, China). ESIMS spectra were recorded on a Shimadzu LCMS-IT-TOF mass spectrometer (Shimadzu, Taiwan). Single-crystal data were measured on an Oxford Gemini S Ultra diffractometer (Oxford Instrument, Oxfordshire, UK). Column chromatography (CC) was performed on silica gel (200–300 mesh, Qingdao Marine Chemical Factory, Qingdao, China) and Sephadex LH-20 (Amersham Pharmacia, Piscataway, NJ, USA).

### 3.2. Fungal Material

The fungus used in this study was isolated from a mangrove, leaves of *S. apetala*, which were collected in Hainan Island, China. The fungus was identified as *Aspergillus* sp. by the ITS region (deposited in GenBank, accession number JX993829). A voucher strain was deposited in the China Center for Type Culture Collection under patent depository number CCTCC M 2012358.

### 3.3. Extraction and Isolation

The fungus *Aspergillus* sp. 16-5c was fermented on autoclaved rice solid-substrate medium for 28 days at room temperature. The mycelia and solid rice medium were extracted with MeOH. Then, the MeOH layer was dried in vacuo to yield 6.8 g of organic extract. The extract was separated by column chromatography (CC) over silica gel eluting with a gradient of CHCl_3_/MeOH from 1:0 to 1:45 to yield five fractions (Fractions 1–5). Fraction 3 (120 mg) was applied to Sephadex LH-20 CC, eluting with CHCl_3_/MeOH (1:1) to obtain Compound **1** (0.8 mg), **2** (1.8 mg), and **3** (2.0 mg). Fraction 4 (65 mg) was applied to Sephadex LH-20, eluting with CHCl_3_/MeOH (1:1) to obtain Compound **4** (1.2 mg), **5** (1.6 mg), and **6** (2.6 mg). Fraction 5 (30 mg) was applied to Sephadex LH-20 CC, eluting with CHCl_3_/MeOH (1:1) to obtain Compound **7** (1.4 mg), **8** (5.6 mg), **9** (2.4 mg), and **10** (4.6 mg).

2-Hydroacetoxydehydroaustin (**1**): colorless crystals (MeOH/CHCl_3_); m.p. >300 °C; [α${]}_{D}^{20}$ = + 60 (*c* 0.01, MeOH); UV (MeOH): λ_max_ 210, 236 nm; IR (KBr): ν_max_ 3528, 2998, 1748, 1431, 1384, 1214 and 1040 cm^−^^1^; ^1^H-NMR(CDCl_3_, 400 MHz), ^13^C-NMR(CDCl_3_, 100 MHz), see Table 1; ESIMS 575[M + H]^+^; HRESIMS *m/z* = 575.2124, [M + H]^+^ (calcd for C_27_H_32_O_8_, 574.2050).

11-Acetoxyisoaustinone (**2**): colorless crystals (MeOH/CHCl_3_); m.p. >300 °C; [α${]}_{D}^{20}$ +150 (*c* 0.1 MeOH); IR (KBr) ν_max_ 3466, 3108, 2988, 2944, 2890, 1754, 1705, 1450, 1377, 1287, and 1226 cm^−1^; ^1^H-NMR (CDCl_3_, 400 MHz) and ^13^C-NMR (CDCl_3_, 100 MHz), see Table 1; ESIMS *m/z* 507 [M + Na]^+^; HRESIMS *m/z* 507.1988 [M + Na]^+^ (calcd for C_27_H_32_O_8_Na, 507.1994).

### 3.4. X-Ray Crystallographic Analysis of ***1***, ***3***, ***4***, ***6***, ***7***, ***8***, ***9*** and ***10***

All single crystal X-ray diffraction data were collected at 150(2) K on an Oxford Gemini S Ultra diffractometer with Cu Kα radiation (λ = 1.54178 Å). The structures were solved by direct methods (SHELXS-97) and refined using full-matrix least-squares difference Fourier techniques. Hydrogen atoms bonded to carbons were placed on the geometrically ideal positions by the “ride on” method. Hydrogen atoms bonded to oxygen were located by the difference Fourier method and were included in the calculation of structure factors with isotropic temperature factors. Crystallographic data for **1**, **3**, **4**, **6**–**10** have been deposited with the Cambridge Crystallographic Data Centre. Copies of the data can be obtained, free of charge, on application to the Director, CCDC 1533328, 12 Union Road, Cambridge CB2 1EZ, UK (fax: t44-(0)1223-336033, or e-mail: deposit@ccdc.cam.ac.uk).

Crystal data of **1**: C_29_H_34_O_12_, Mr = 574.56, Orthorhombic, a = 8.8330(2) Å, b = 12.2482(2) Å, c = 12.3432(2) Å, α = 90°, β = 97.342(2)°, γ = 90°, V =1324.44(4) Å3, space group P21, Z = 2, Dx = 1.363 mg/m^3^, μ (Cu Kα) = 0.850 mm^−1^, and F (000) = 1064. Crystal dimensions: 0.40 × 0.33 × 0.29 mm. Independent reflections: 4336 (Rint = 0.0392). The final R1 values were 0.0258, wR2 = 0.0659 (I > 2σ(I)). Flack parameter = 0.08 (8). CCDC number: 1533329.

Crystal data of **3**: C_25_H_30_O_6_, Mr = 426.49, Monoclinic, a = 11.5899(2) Å, b = 7.4432(2) Å, c = 12.0804(2) Å, α = 90°, β = 92.060(2)°, γ = 90°, V = 1041.45(4) Å3, space group P21, Z = 2, Dx = 1.360 mg/m^3^, μ(Cu Kα) = 0.786 mm^−1^, and F (000) = 456. Crystal dimensions: 0.30 × 0.23 × 0.22 mm. Independent reflections: 3696 (Rint = 0.0549). The final R1 values were 0.0476, wR2 = 0.1215 (I > 2σ(I)). Flack parameter = 0.01 (2). CCDC number: 921711.

Crystal data of **4**: C_27_H_32_O_9_, Mr = 500.53, Orthorhombic, a = 7.71560(10) Å, b = 14.9523(2) Å, c = 21.1376(3) Å, α = 90°, β = 90°, γ = 90°, V = 2438.56(6) Å3, space group P212121, Z = 4, Dx = 1.363 mg/m^3^, μ (Cu Kα) = 0.850 mm^−1^, and F (000) = 1064. Crystal dimensions: 0.40 mm × 0.33 mm × 0.29 mm. Independent reflections: 4336 (Rint = 0.0392). The final R1 values were 0.0258, wR2 = 0.0659 (I > 2σ(I)). Flack parameter = 0.08 (12). CCDC number: 921707.

Crystal data of **6**: C_29_H_32_O_11_, Mr = 556.55, Monoclinic, a = 8.8841 (3) Å, b = 12.5166 (4) Å, c = 12.1254 (5) Å, α = 90°, β = 99.052 (4) °, γ = 90°, V = 1331.54 (8) Å3, space group P21, Z = 2, Dx = 1.388 mg/m^3^, μ (Cu Kα) = 0.897 mm^−1^, and F (000) = 588. Crystal dimensions: 0.44 mm × 0.35 mm × 0.21 mm. Independent reflections: 4474 (Rint = 0.0620). The final R1 values were 0.0326, wR2 = 0.0740 (I > 2σ (I)). Flack parameter = 0.01 (1). CCDC number: 921708.

Crystal data of **7**: C_29_H_33_NO_9_, Mr = 539.56, Hexagonal, a = 11.20730 (10) Å, b = 11.20730 (10) Å, c = 36.3299(5) Å, α = 90°, β = 90°, γ = 120°, V = 3951.82(7) Å3, space group P65, Z = 6, Dx = 1.360 mg/m^3^, μ(Cu Kα) = 0.841 mm^−1^, and F (000) = 1716. Crystal dimensions: 0.44 mm × 0.35 mm × 0.32 mm. Independent reflections: 4596 (Rint = 0.0815). The final R1 values were 0.0597, wR2 = 0.1612 (I > 2σ(I)). Flack parameter = 0.01 (1). CCDC number: 921709.

Crystal data of **8**: C_25_H_28_O_8_, Mr = 456.47, Orthorhombic, a = 7.8409(2) Å, b = 11.6017(2) Å, c = 23.9529(4) Å, α = 90°, β = 90°, γ = 90°, V = 2178.94(8) Å3, space group P212121, Z = 4, Dx = 1.391 mg/m^3^, μ (Cu Kα) = 0.862 mm^−1^, and F (000) = 968. Crystal dimensions: 0.37 mm × 0.31 mm × 0.22 mm. Independent reflections: 3880 (Rint = 0.0455). The final R1 values were 0.0277, wR2 = 0.0684 (I > 2σ(I)). Flack parameter = 0.01 (2). CCDC number: 921710.

Crystal data of **9**: C_26_H_34_O_7_, Mr = 458.53, Orthorhombic, a = 8.29600(10) Å, b = 13.9817(2) Å, c = 19.5230(2) Å, α = 90°, β = 90°, γ = 90°, V = 2264.52(5) Å3, space group P212121, Z = 4, Dx = 1.345 mg/m^3^, μ (Cu Kα) = 0.793 mm^−1^, and F (000) = 984. Crystal dimensions: 0.33 mm × 0.30 mm × 0.25 mm. Independent reflections: 4027 (Rint = 0.0434). The final R1 values were 0.0280, wR2 = 0.0728 (I > 2σ (I)). Flack parameter = 0.08 (12). CCDC number: 921712.

Crystal data of **10**: C_29_H_34_O_11_, Mr = 558.56, Orthorhombic, a = 8.29600(10) Å, b = 13.9817(2) Å, c = 19.5230(2) Å, α = 90°, β = 98.7090(10)°, γ = 90°, V = 2264.52(5) Å3, space group P21, Z = 2, Dx = 1.345 mg/m^3^, μ (Cu Kα) = 0.793 mm^−1^, and F (000) = 984. Crystal dimensions: 0.33 mm × 0.30 mm × 0.25 mm. Independent reflections: 4027 (Rint = 0.0434). The final R1 values were 0.0280, wR2 = 0.0728 (I > 2σ (I)). Flack parameter = 0.04 (11). CCDC number: 1533328.

### 3.5. Assays for Enzyme Inhibiting Activities and Cytotoxic Activities

These two experiments were conducted according to reference procedures [14,23].

## 4. Conclusions

One new and nine known meroterpenoids (**1**–**10**) were isolated and identified from the culture of the endophytic fungus *Aspergillus* sp. 16-5c. The absolute configurations of Known Compounds **3**, **7**, **8**, and **9** were first identified. Compounds **3**, **7**, and **8** showed acetylcholinesterase (AchE) inhibitory activity with IC_50_ values of 2.50, 0.40, and 3.00 μM, respectively.

## Figures and Tables

**Table 1:** ^1^H (400 MHz) and ^13^C (100 MHz) NMR data for **1** and **2** (CDCl_3_, in ppm).

Position	1		2	
	δ_H_ (*J* in Hz)	δ_C_	δ_H_ (*J* in Hz)	δ_C_
1	2.23, dd (11.5, 13.4)	37.1, CH_2_	6.52, d (9.9)	146.7, CH
	2.98, dd (7.4, 13.4)			
2	4.28, dd (7.4, 11.5)	64.8, CH	6.03, d (9.9)	120.0, CH
3		174.9, C		163.7, C
4		90.0, C		85.6, C
5		46.1, C		46.3, C
6	1.76, dd (11.9, 13.0)	36.1, CH_2_	2.66, td (13.5, 3.6)	27.1, CH_2_
	1.90, dd (3.9, 13.0)		1.79, dt (13.5, 3.6)	
7	5.35, dd (3.9, 11.9)	67.8, CH	1.67,dd (12.2, 3.6)	27.0, CH_2_
			1.61,dd (12.2, 3.6)	
8		56.6, C		41.3, C
9		93.2, C		134.8, C
10		138.1, C		138.1, C
11	5.70, s	74.3, CH	5.74, s	74.1, CH
12	1.37, s	12.0, CH_3_	1.57, s	23.0, CH_3_
13	5.81, d (1.5)	124.4, CH_2_	1.75, s	15.2, CH_3_
	5.85, d (1.5)			
14	1.43, s	23.5, CH_3_	1.37, s	25.9, CH_3_
15	1.47, s	27.4, CH_3_	1.20, s	22.8, CH_3_
16		170.6, C	2.04, s	20.7, CH_3_
17	2.07, s	20.8, CH_3_		171.5, C
18		168.9, C		
19	2.05, s	21.1, CH_3_		
1′	5.73, s	116.9, CH_2_	5.41, d (0.9)	111.7, CH
	6.14, s		5.33, d (0.9)	
2′		136.9, C		142.6, C
3′		82.5, C		59.2, C
4′		168.9, C		209.5, C
5′	5.26, q (6.9)	76.6 d	4.35, q (6.4)	76.2, CH
6′		85.5, C		91.2, C
7′		61.6, C		66.4, C
8′		167.3, C		169.1, C
9′	1.58, s	19.6, CH_3_	1.25, s	12.9, CH_3_
10′	1.70, d (6.9)	13.8, CH_3_	1.30, d (6.4)	12.7, CH_3_

**Table 2:** Acetylcholinesterase (AChE) inhibitory activity of Compounds **1**–**10** ^a^.

Compound	1	2	3	4	5	6	7	8	9	10	HUP ^b^
	>50	>50	2.50	>50	>50	>50	0.40	3.00	>50	>50	0.07

^a^ Data are expressed in IC_50_ values (μmol/L). ^b^ HUP (Huperzine A) used as positive control.

## Supplementary Materials

The following are available online, Figure S1: The NMR spectrum of compounds **1**–**10**, and the perspective ORTEP drawings for compounds **1**, **3**, **4**, **6**, **7**, **8**, **9** and **10**.
