# Supplementary material for: Acetylcholinesterase Inhibitory Meroterpenoid from a Mangrove Endophytic Fungus Aspergillus sp. 16-5c
Source: Molecules. 2017 May 3;22(5):727. doi: 10.3390/molecules22050727 (PMC6154586; doi:10.3390/molecules22050727)
Supplement: Supplementary file 1 [file molecules-22-00727-s001.pdf]

Supporting information

Acetylcholinesterase Inhibitory Meroterpenoids from a Mangrove Endophytic Fungus *Aspergillus* sp. 16-5c

Yuhua Long [1, 2], Hui Cui[1], Xinglie Liu[3], Ze'en Xiao[1], Shitong Wen[2], Zhigang She[1], and Xishan Huang [1*]

[1] School of Chemistry, Sun Yat-Sen University, Guangzhou510275, China; E-Mails: yuhualong68@hotmail.com (Y.L.); cuihui2@mail2.sysu.edu.cn (H.C.); 383037800@qq.com (Z.X.); huangxishan13@foxmail.com (X.H.).

[2] School of Chemistry and Environment, South China Normal University, 348 West Outer Ring Road, Guangzhou 510006, China; E-Mails: yuhualong68@hotmail.com (Y.L.).

[3] The Sixth Affiliated Hospital of Sun Yat-sen University, Guangzhou 510275, China; E-Mails: 13922342800@163.com (X.L.);

* Correspondence: E-Mails: huangxishan13@foxmail.com (X.H.); Tel./ Fax: +86-20-8411-0392.

Received: date; Accepted: date; Published: date

List of Supporting Information

Figure S1. The NMR spectrum of Compounds **1**–**10**……………………………..……………S1

Figure S2. The perspective ORTEP drawings for Compounds **1, 3, 4, 6, 7, 8** and **10**…………S29

Figure S3. The ESIMS or HRESIMS and HREIMS of Compounds **1** and **2**…………………S33

The ^{1}H-NMR (400 MHz) spectrum of Compound 1 in $CDCl_3$

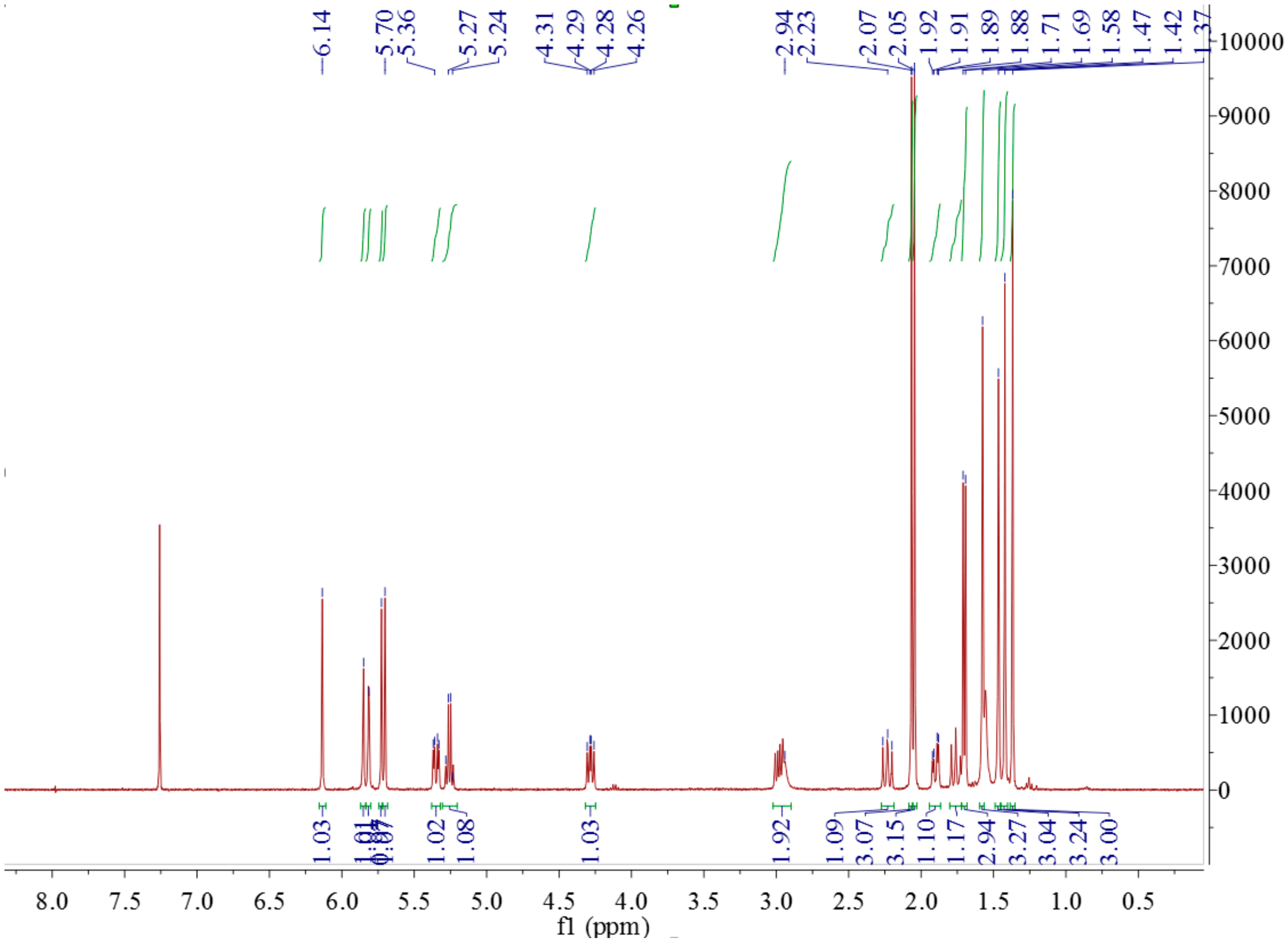

The ^{13}C-NMR (400 MHz) spectrum of Compound 1 in $CDCl_3$

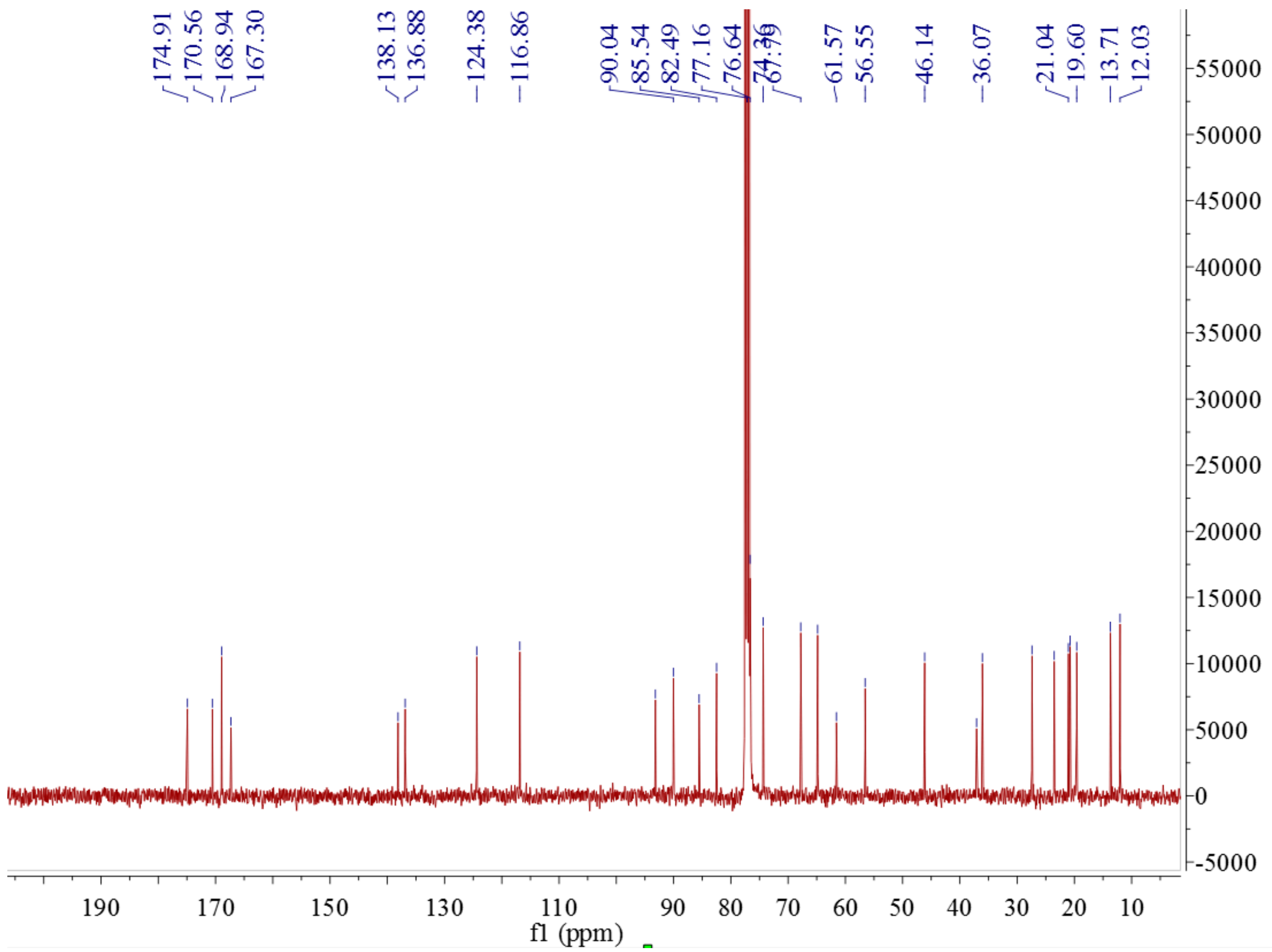

The DEPT-135 (400 MHz) spectrum of Compound 1 in $CDCl_3$

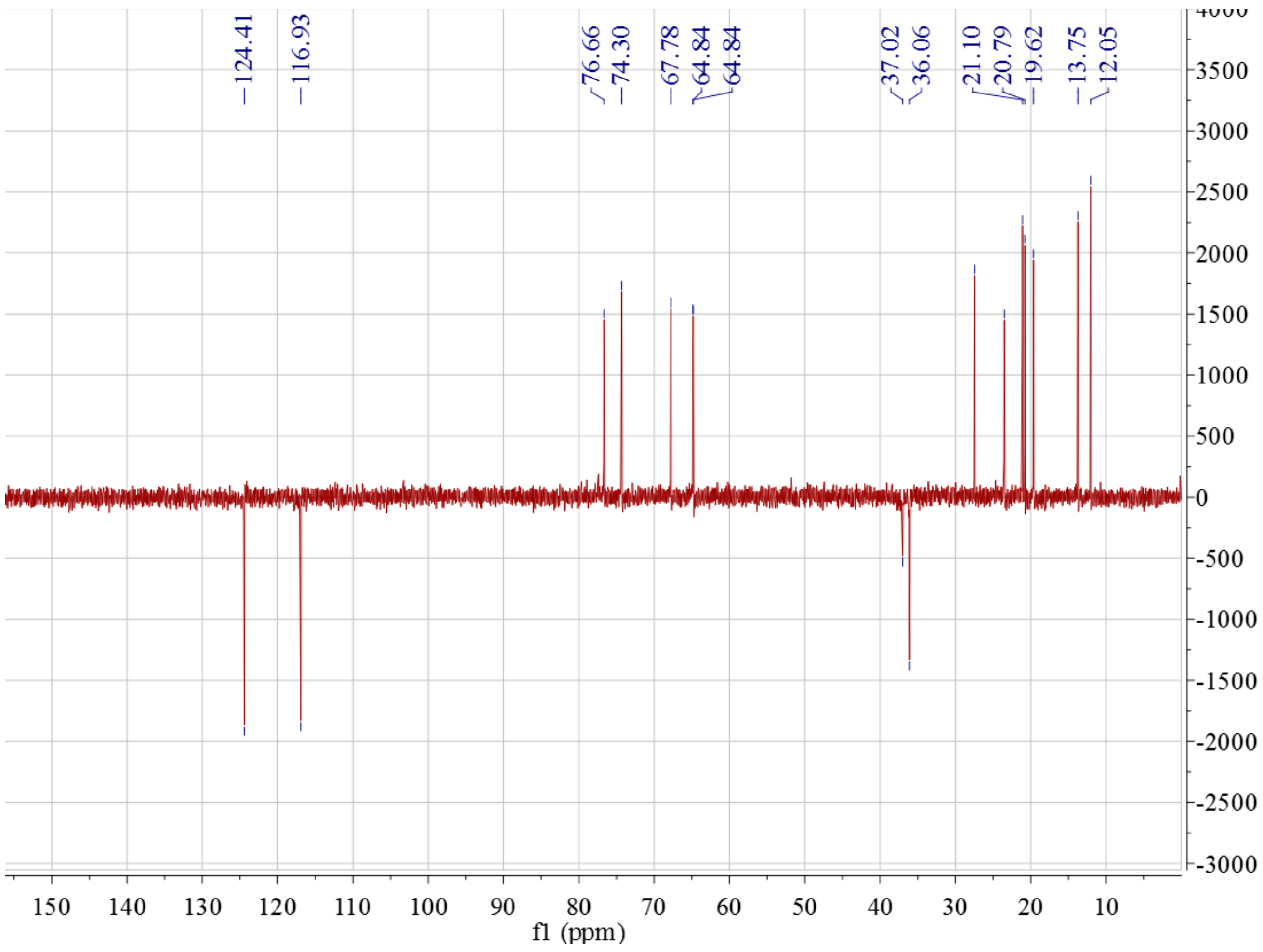

The ^{1}H-^{1}HCOSY (400 MHz) spectrum of Compound 1 in $CDCl_3$

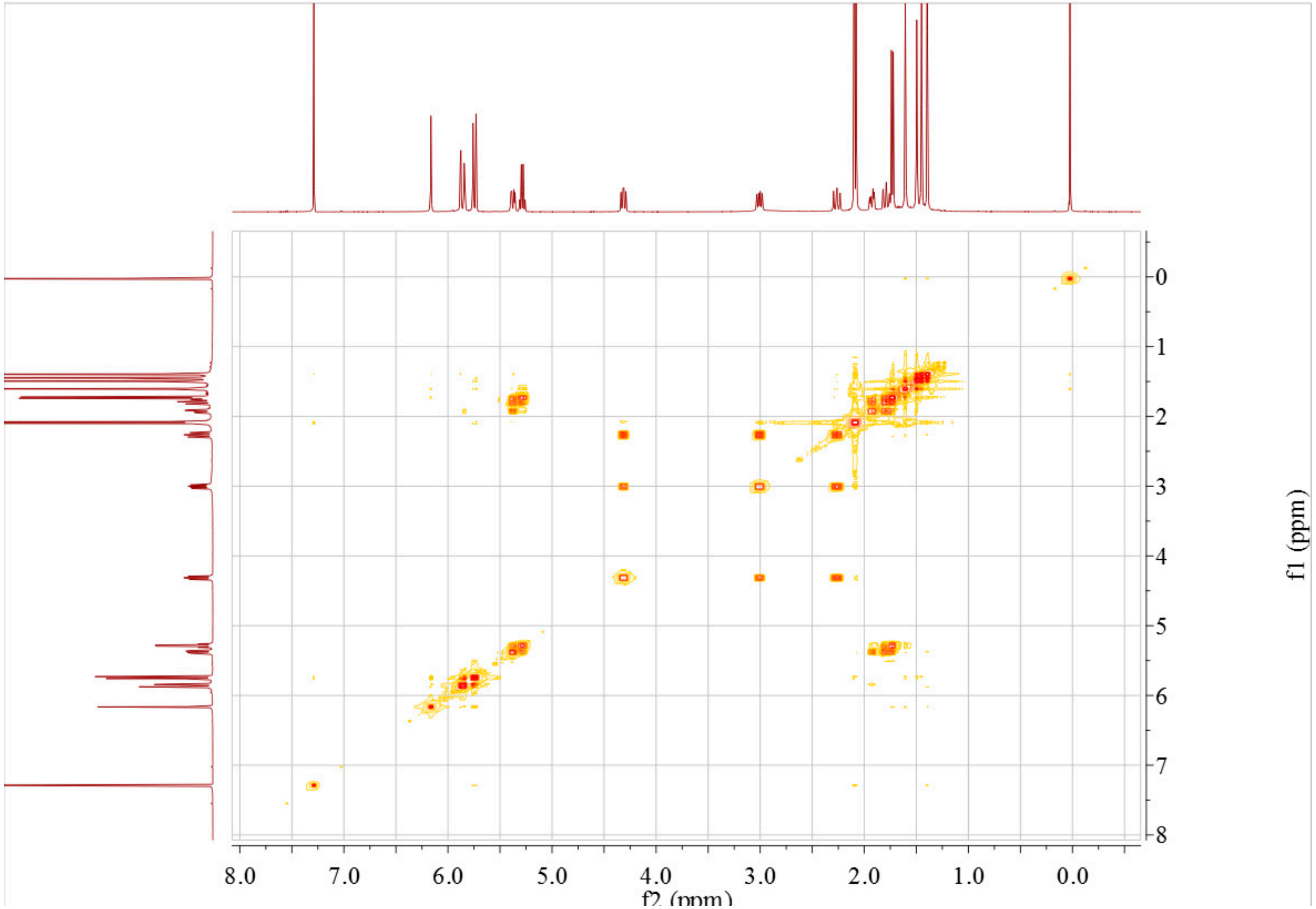

The HSQC (400 MHz) spectrum of Compound 1 in $CDCl_3$

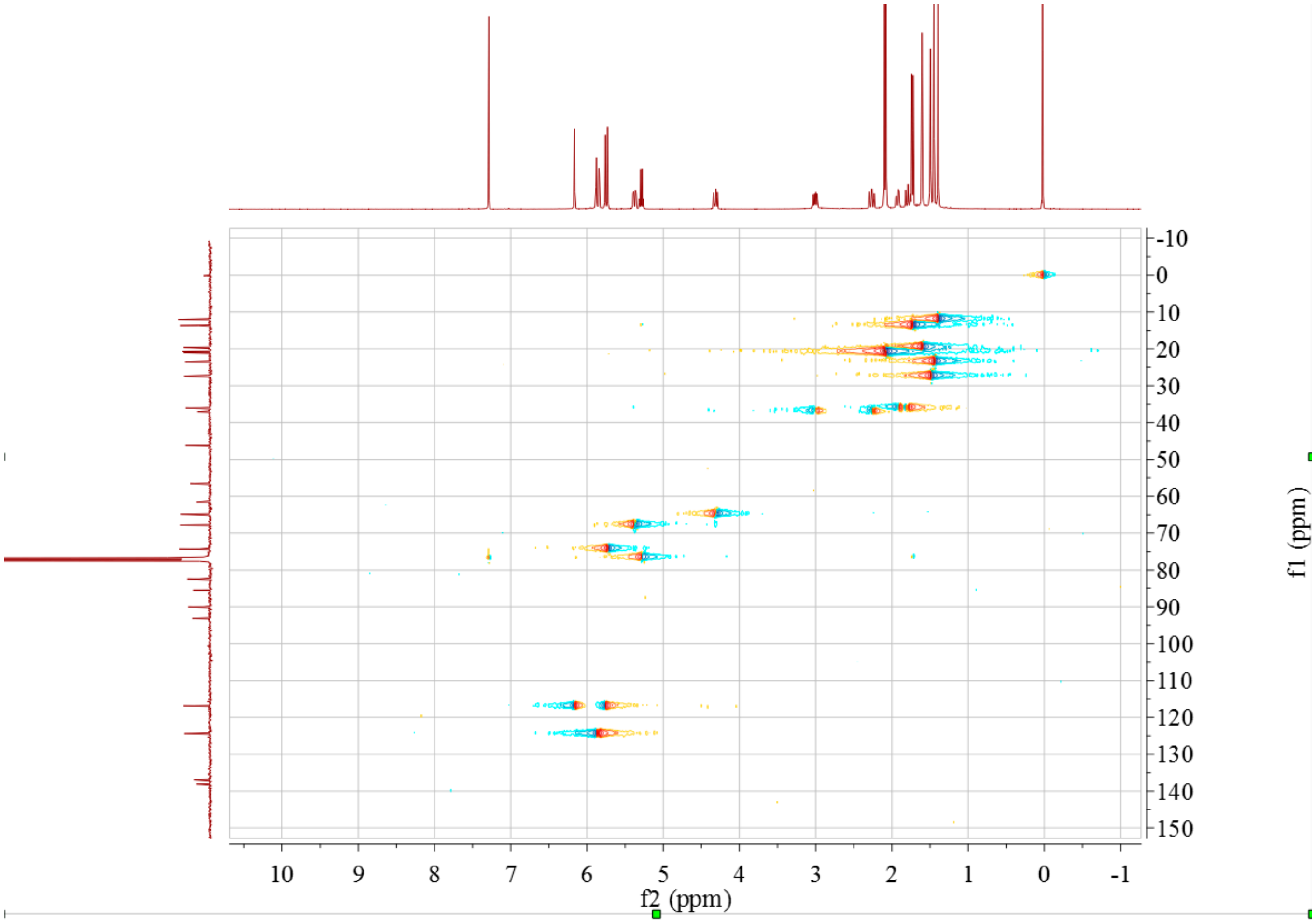

The HMBC (400 MHz) spectrum of Compound 1 in $CDCl_3$

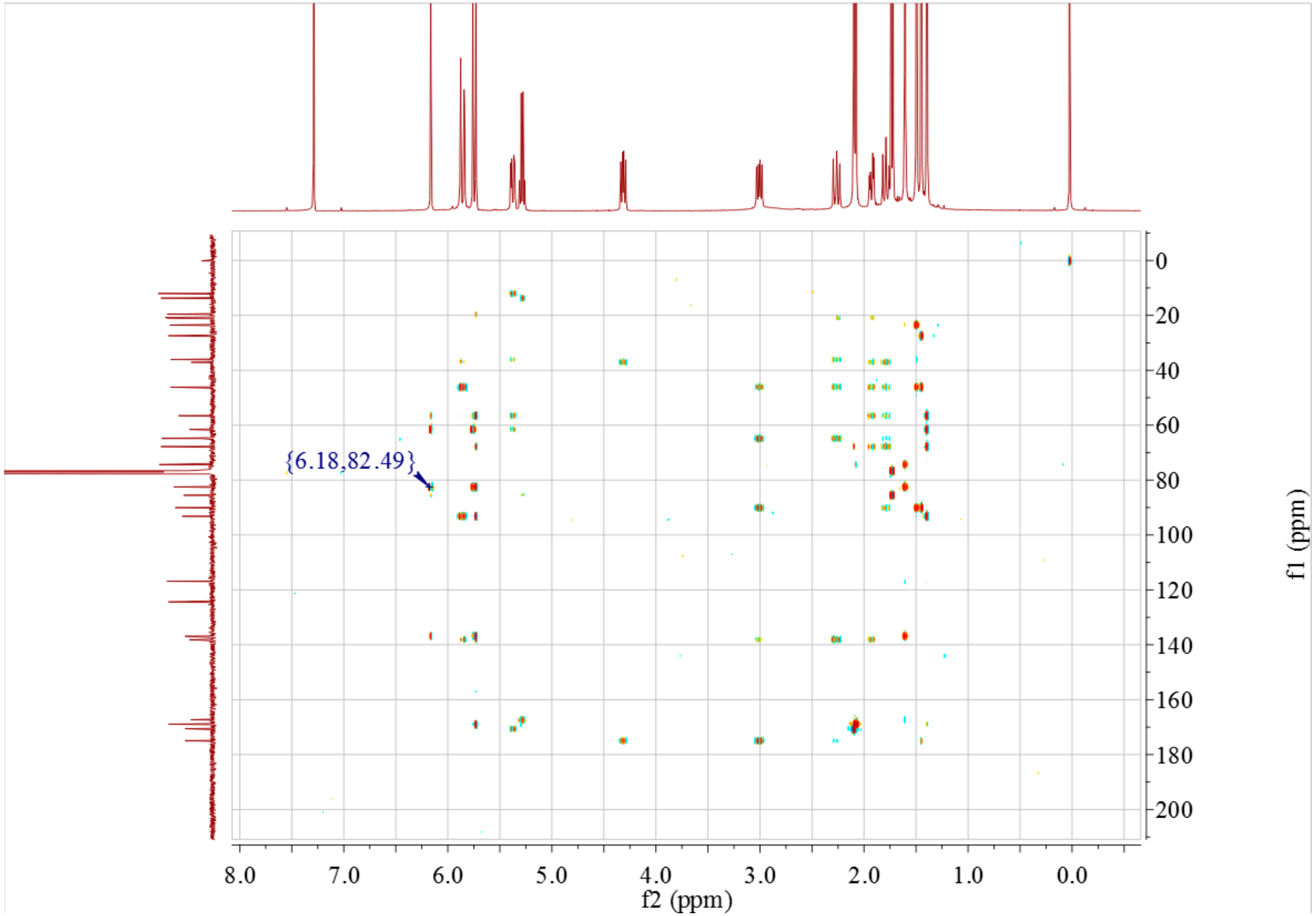

The ^{1}H-NMR (400 MHz) spectrum of Compound 2 in $CDCl_3$

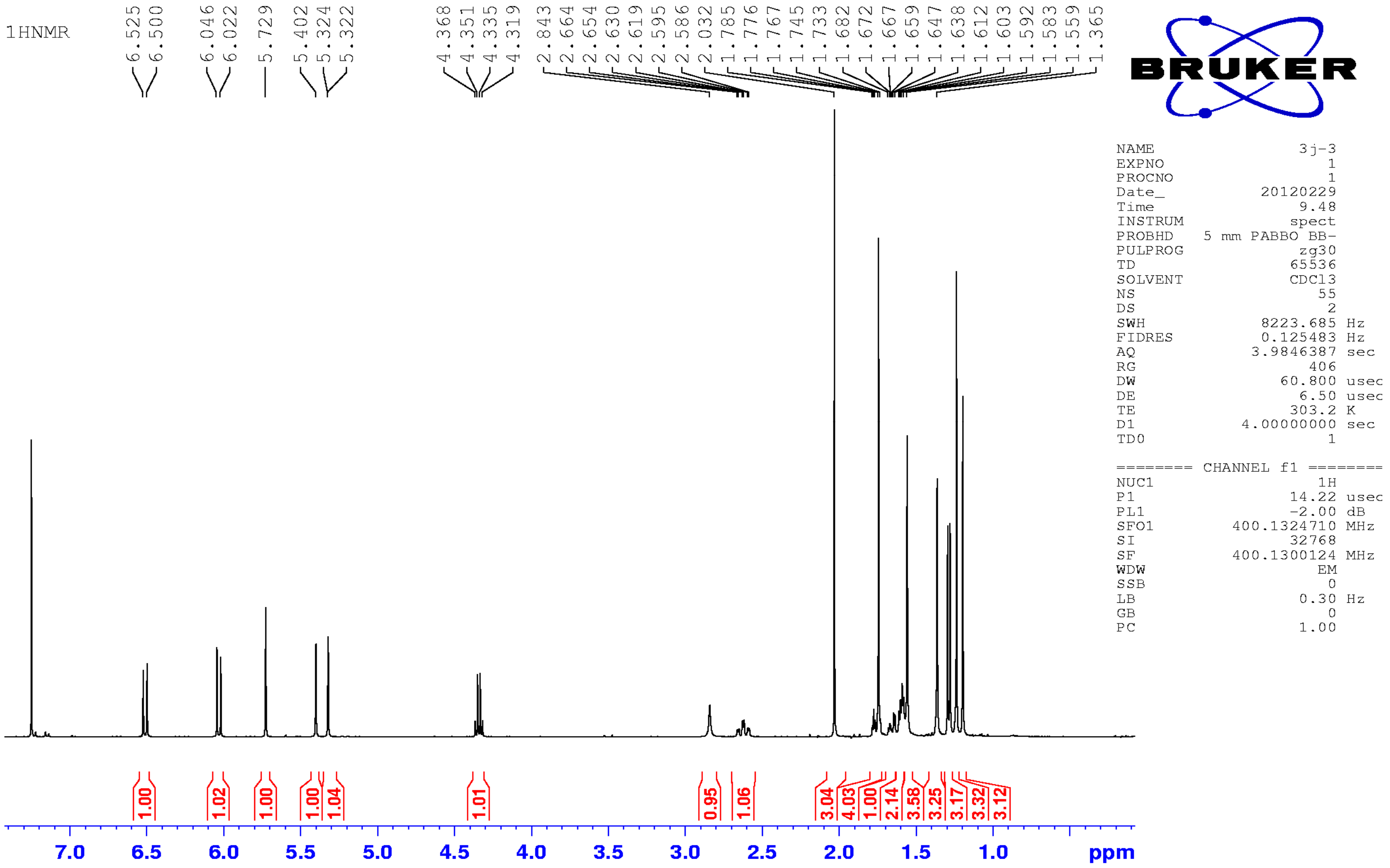

The ^{13}C-NMR (400 MHz) spectrum of Compound 2 in $CDCl_3$

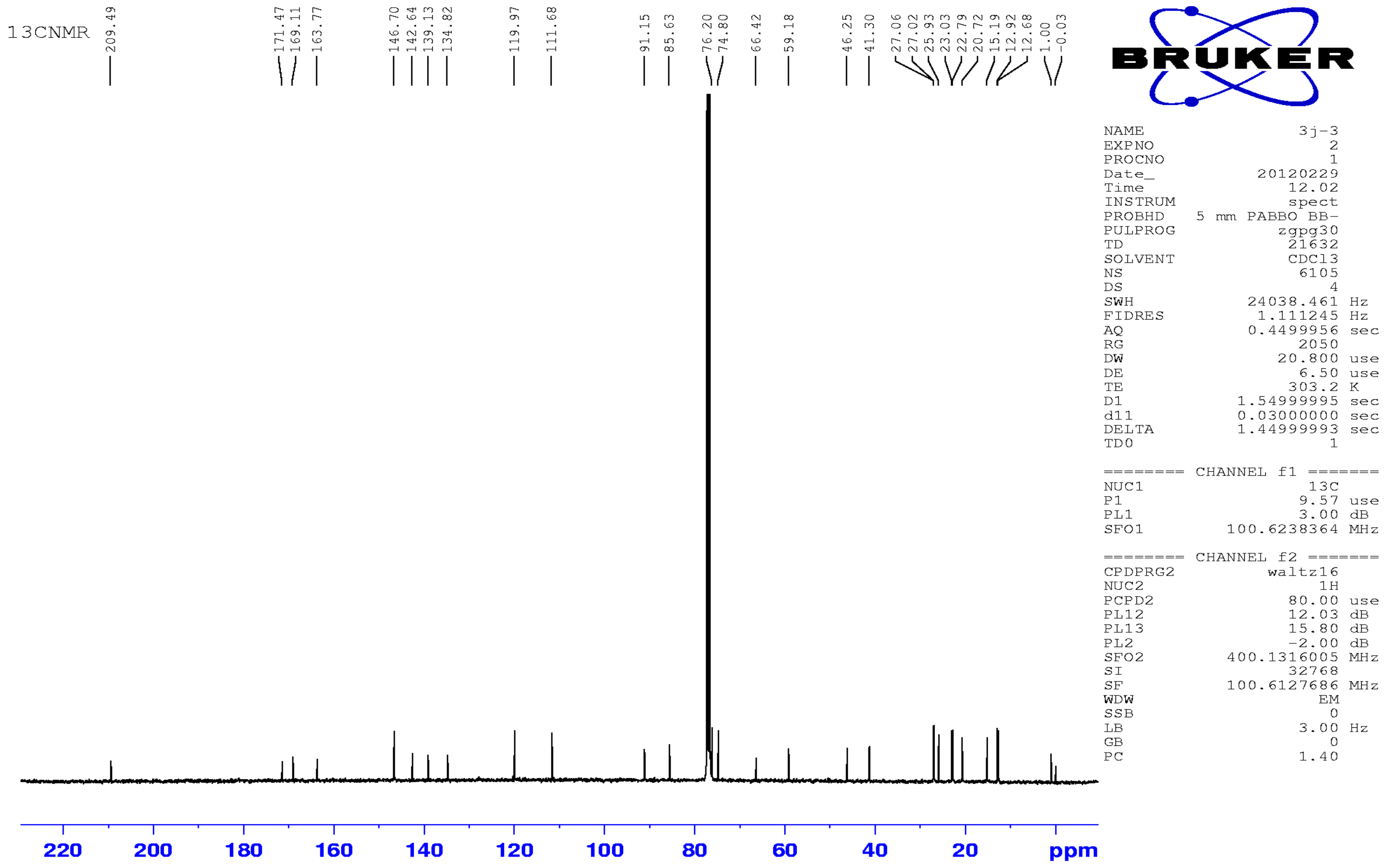

The ^{1}H- ^{1}H COSY (400 MHz) spectrum of Compound 2 in $CDCl_3$

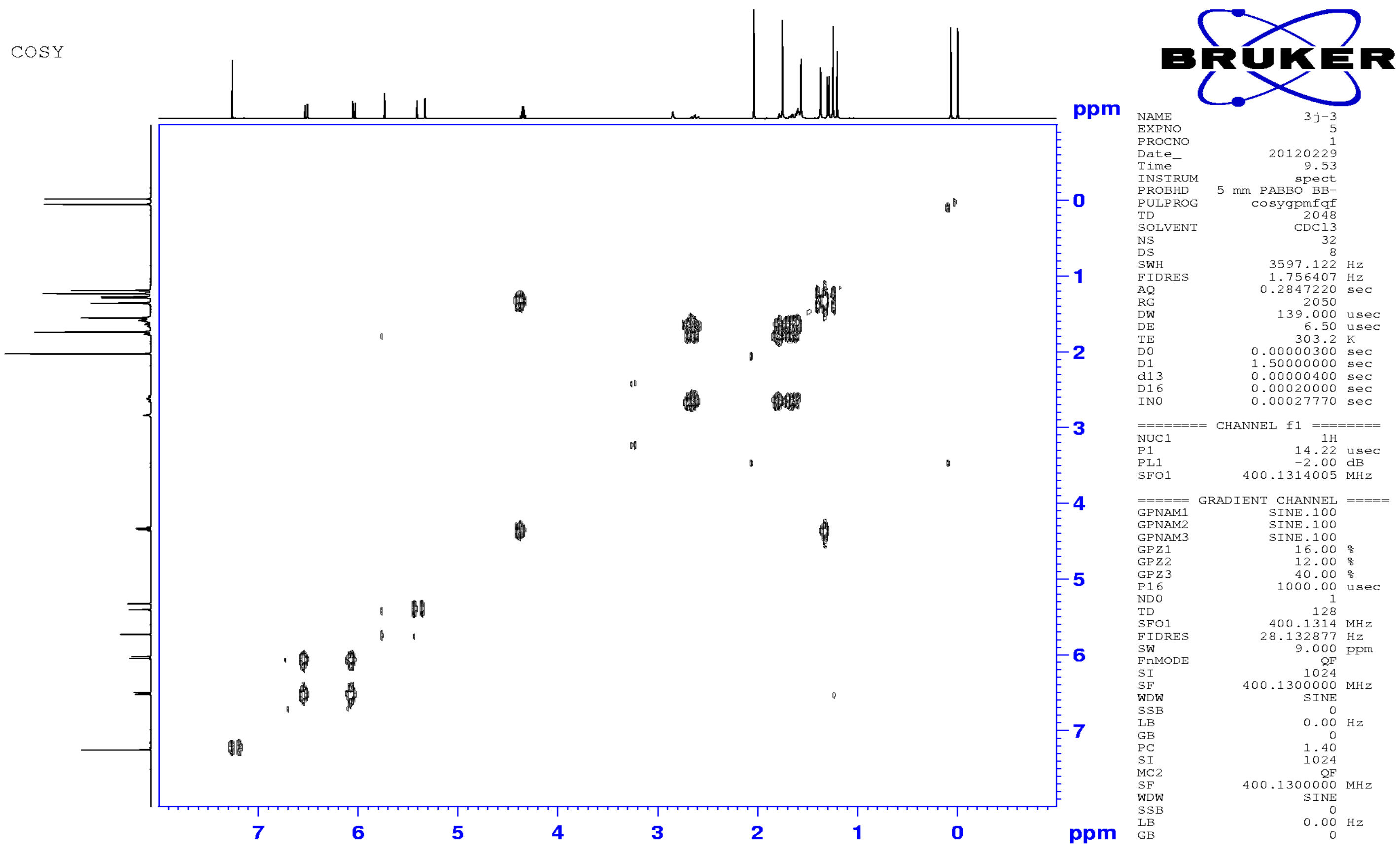

The HSQC (400 MHz) spectrum of Compound 2 in $CDCl_3$

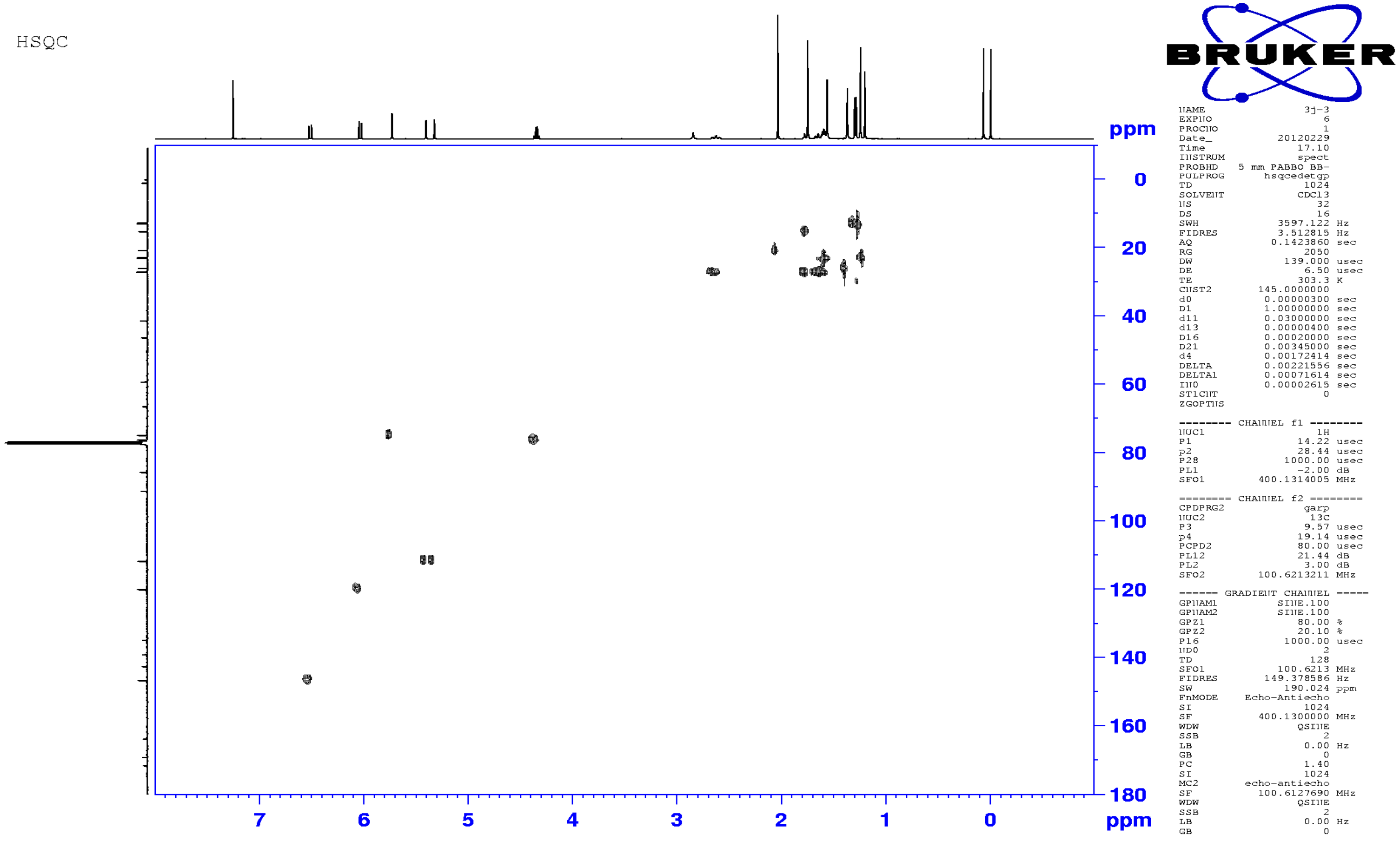

The HMBC (400 MHz) spectrum of Compound 2 in $CDCl_3$

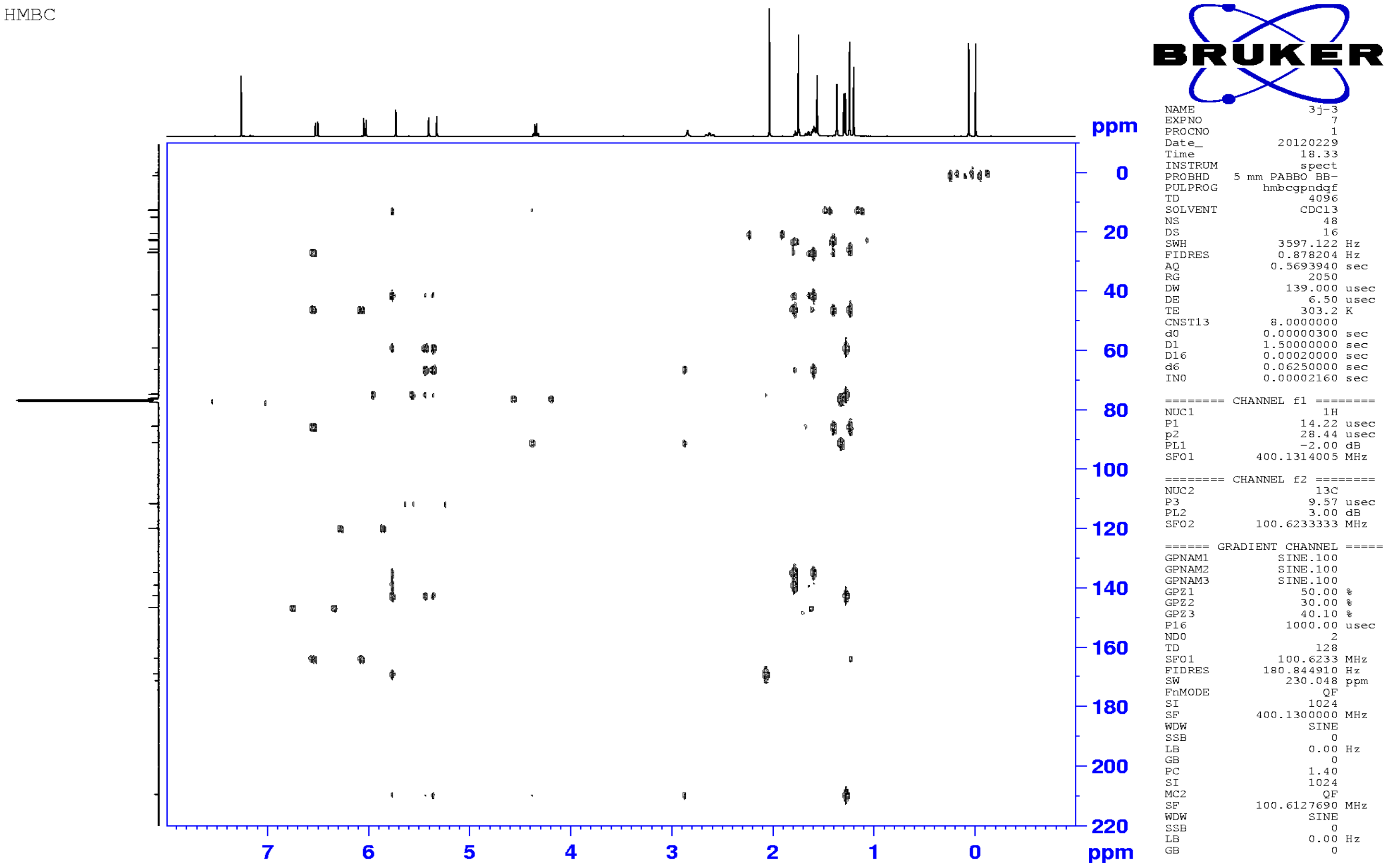

The NOE (400 MHz) spectrum of Compound 2 in $CDCl_3$

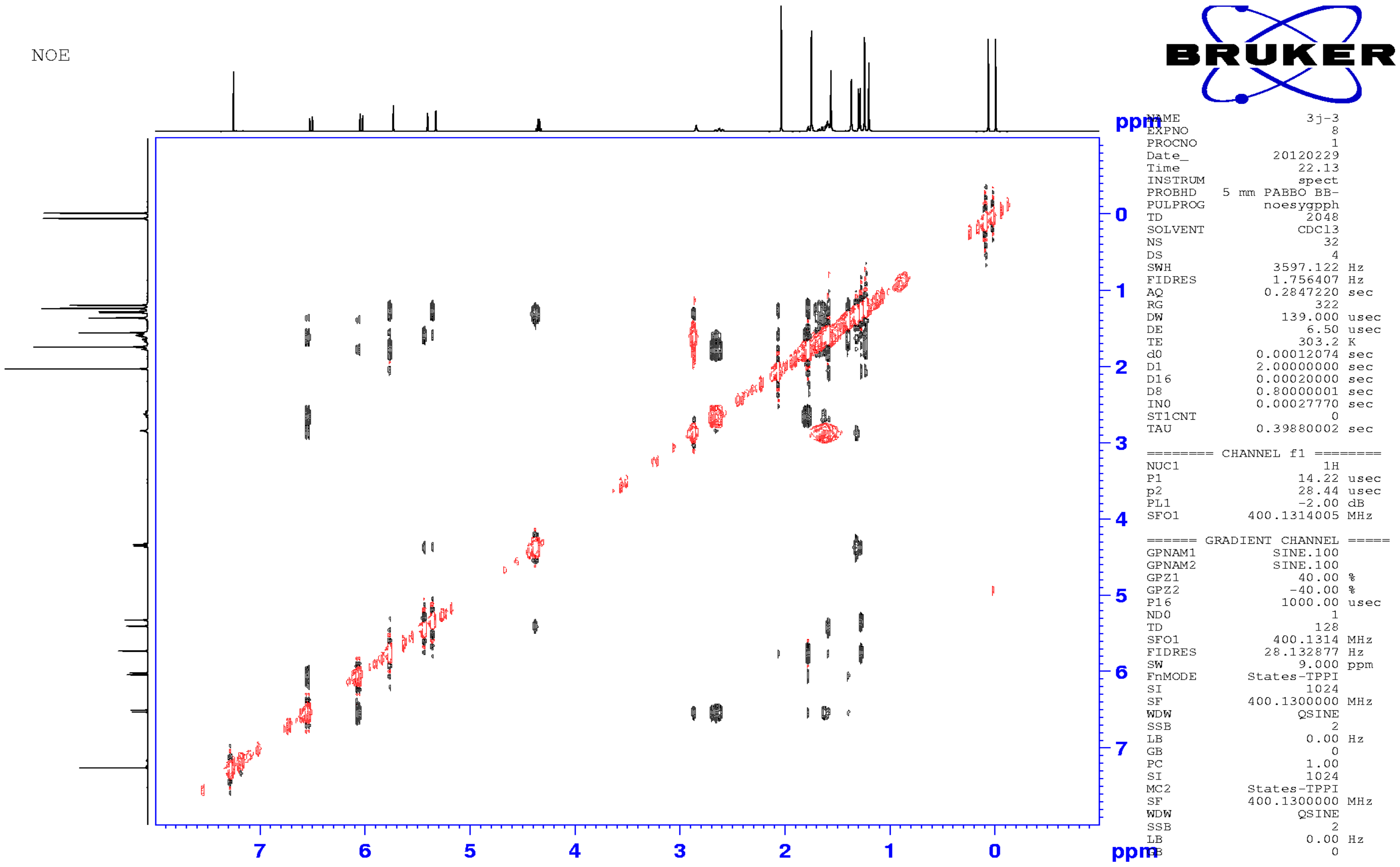

The ^{1}H-NMR (400 MHz) spectrum of Compound 3 in $CDCl_3$

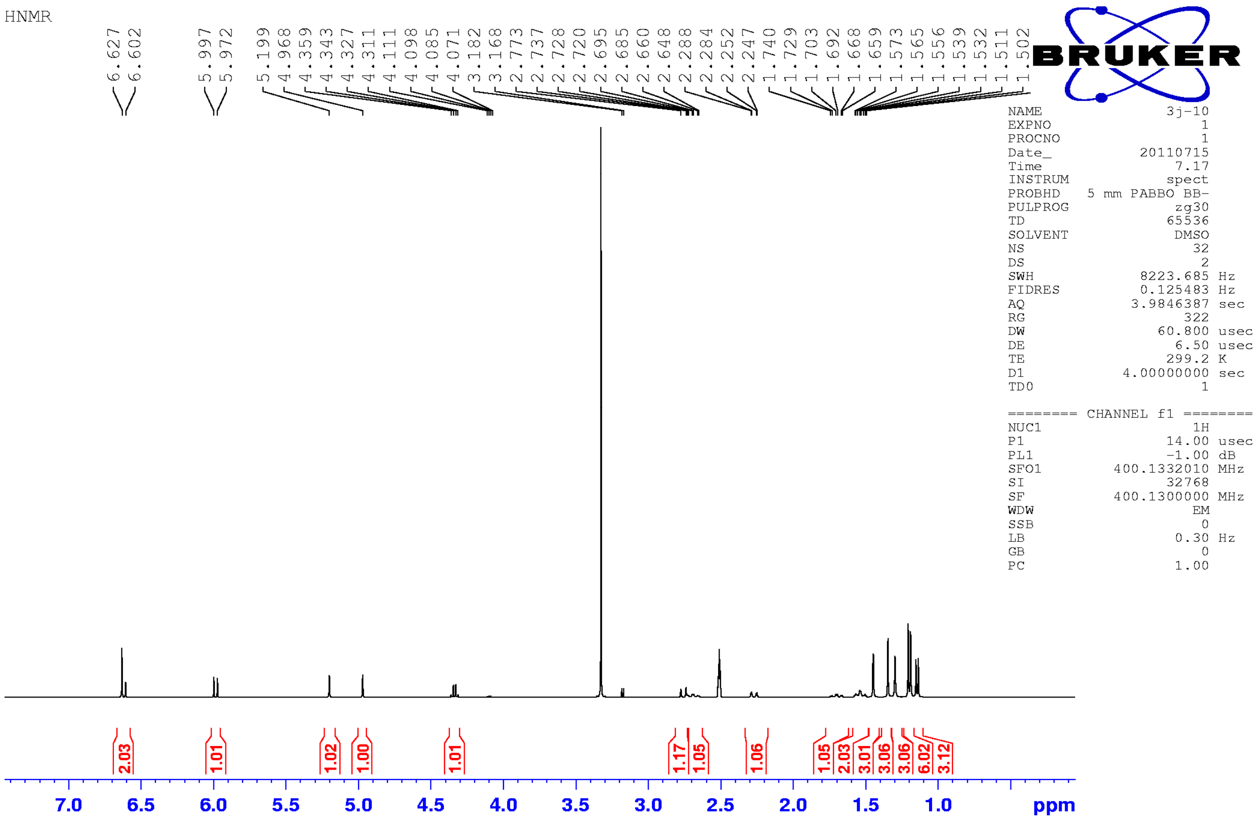

The ^{13}C-NMR (400 MHz) spectrum of Compound 3 in $CDCl_3$

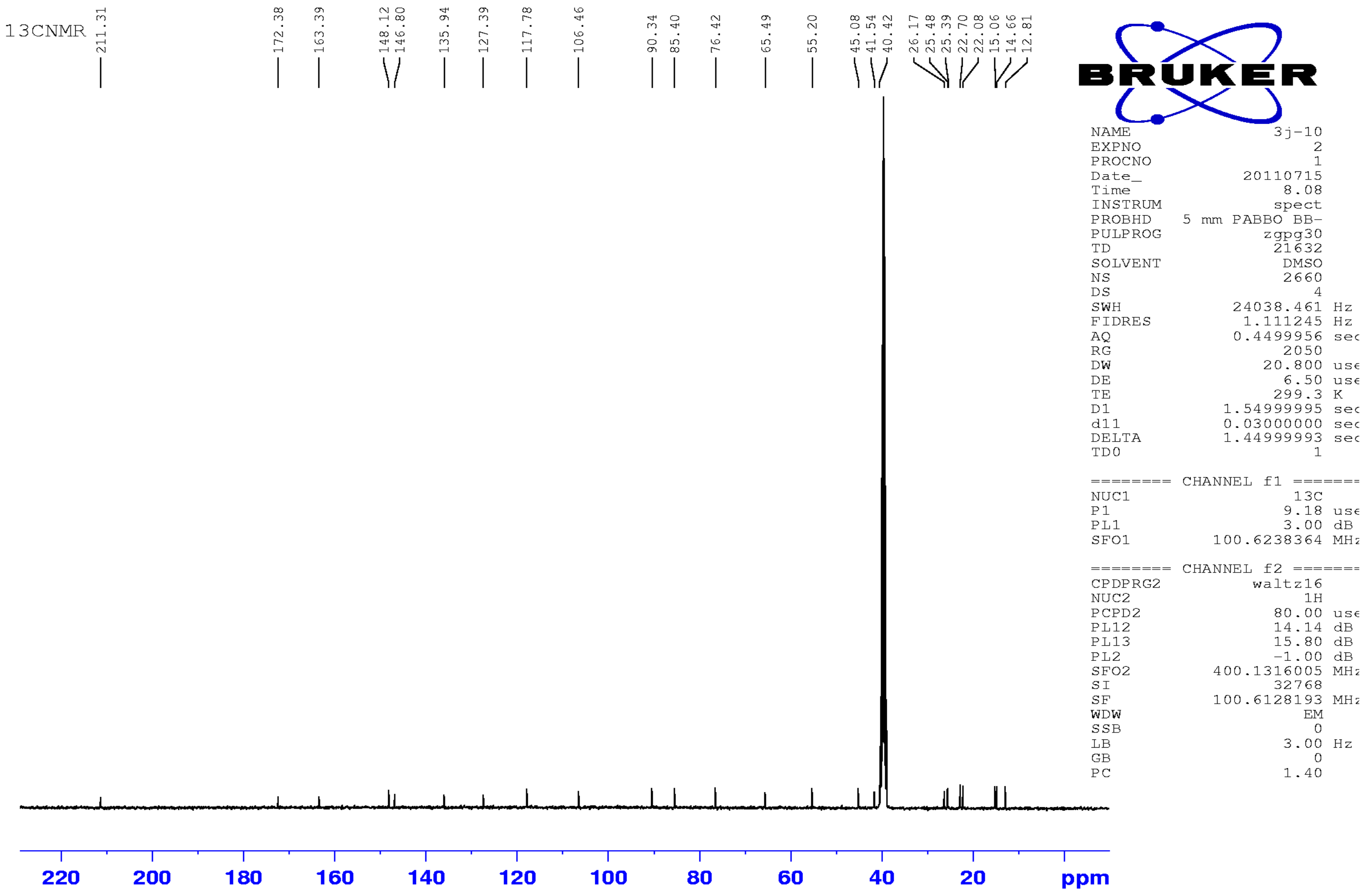

The ^{1}H-NMR (400 MHz) spectrum of Compound 4 in $CDCl_3$

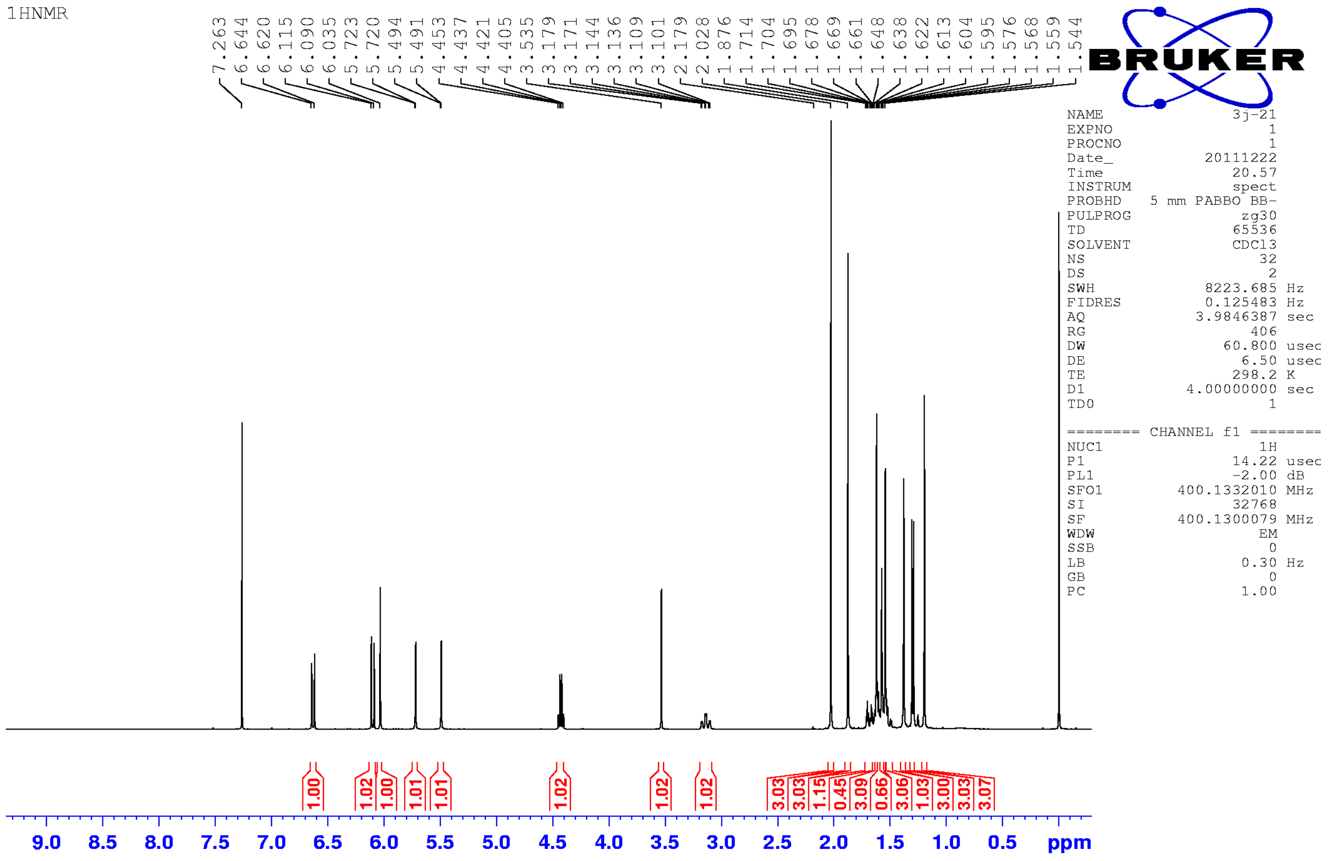

The ^{13}C-NMR (400 MHz) spectrum of Compound 4 in $CDCl_3$

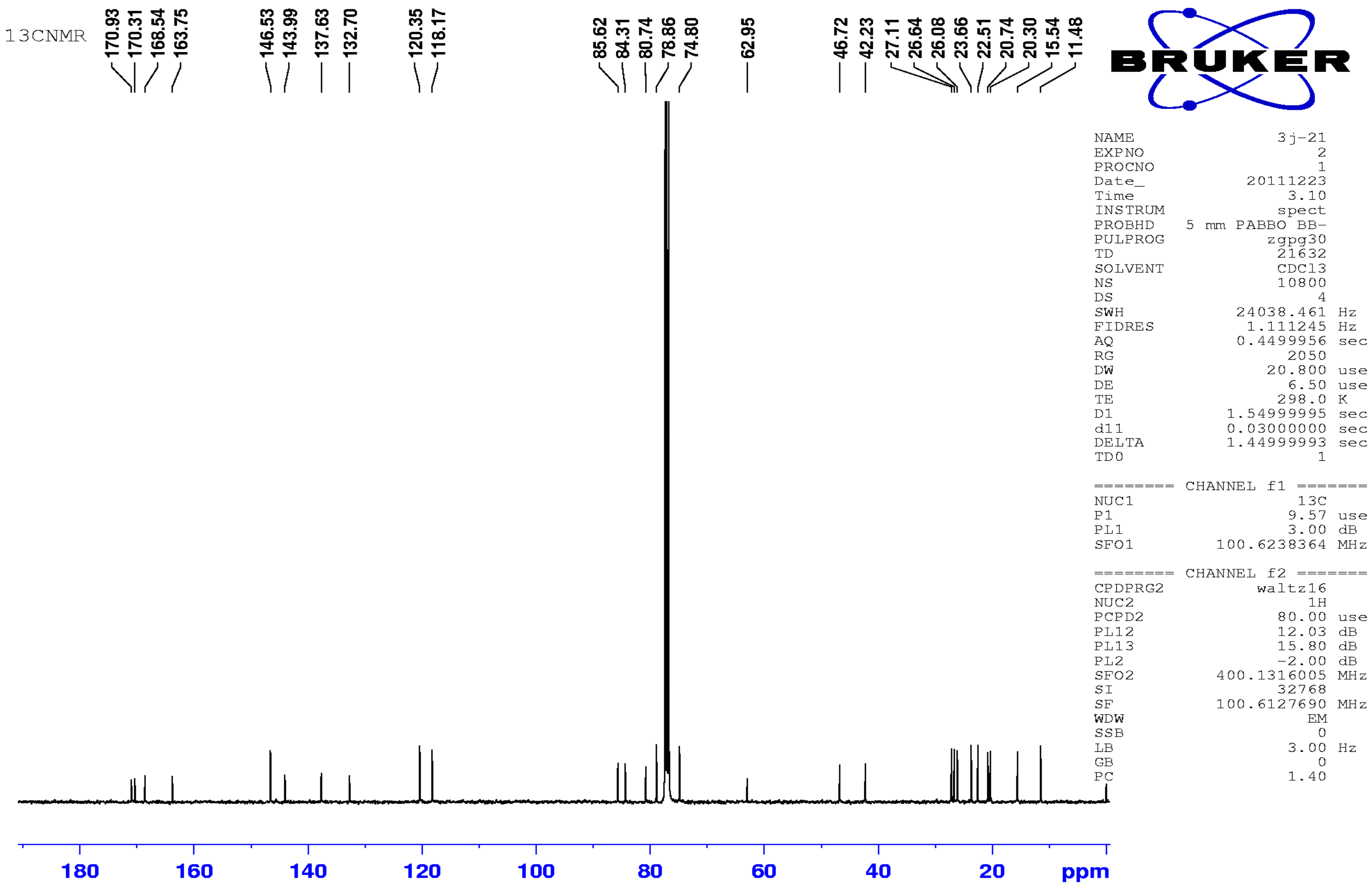


```
NAME            3j-21
EXPNO               2
PROCNO              1
Date_        20111223
Time             3.10
INSTRUM         spect
PROBHD   5 mm PABBO BB-
PULPROG       zgpg30
TD              21632
SOLVENT         CDCl3
NS              10800
DS                  4
SWH         24038.461 Hz
FIDRES       1.111245 Hz
AQ          0.4499956 sec
RG               2050
DW             20.800 use
DE               6.50 use
TE              298.0 K
D1         1.54999995 sec
d11        0.03000000 sec
DELTA      1.44999993 sec
TD0                 1

======== CHANNEL f1 =======
NUC1              13C
P1               9.57 use
PL1              3.00 dB
SFO1      100.6238364 MHz

======== CHANNEL f2 =======
CPDPRG2       waltz16
NUC2               1H
PCPD2           80.00 use
PL12            12.03 dB
PL13            15.80 dB
PL2             -2.00 dB
SFO2      400.1316005 MHz
SI              32768
SF        100.6127690 MHz
WDW                EM
SSB                 0
LB               3.00 Hz
GB                  0
PC               1.40
```

The ¹H-NMR (400 MHz) spectrum of Compound 5 in DMSO

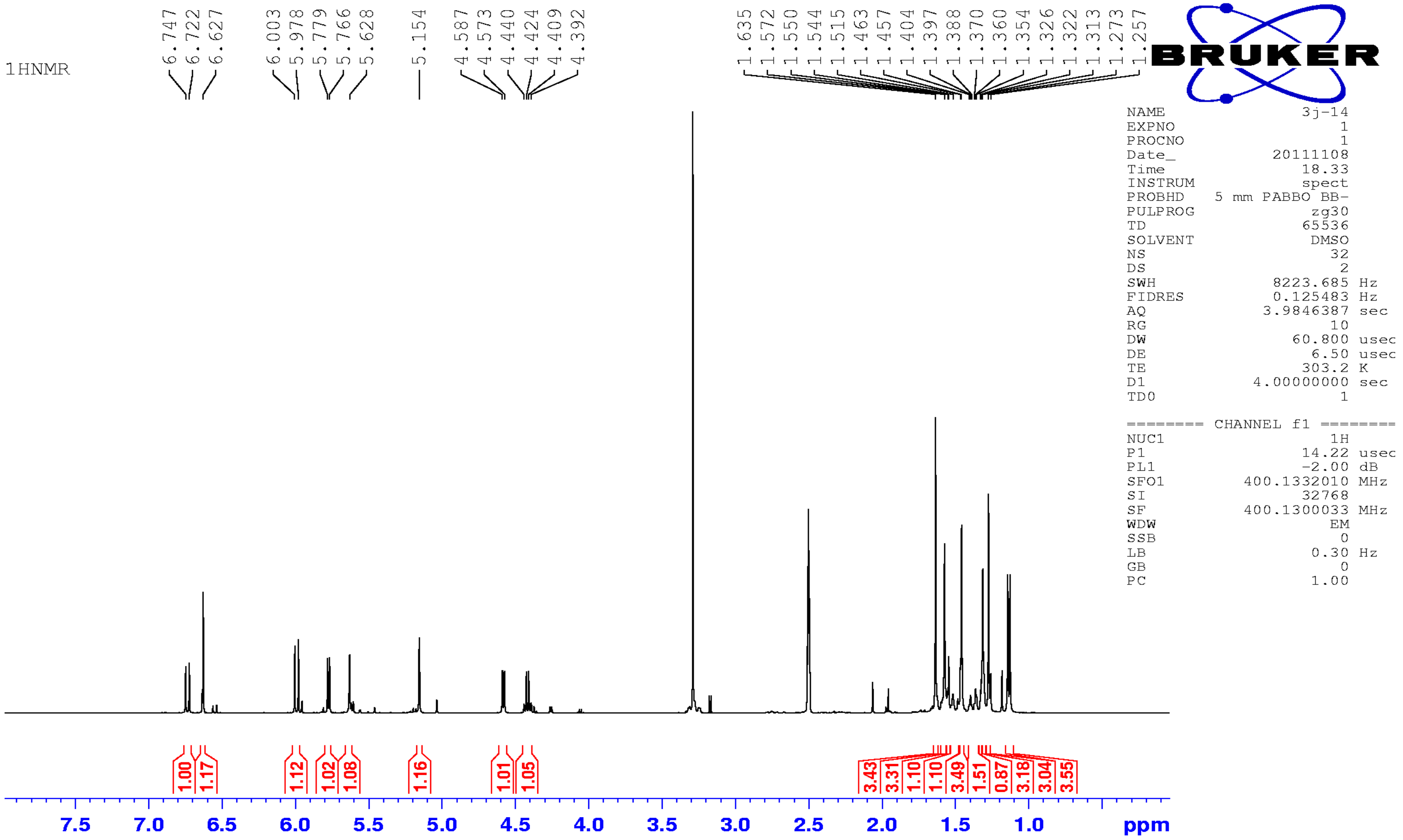

The ^{13}C-NMR (400 MHz) spectrum of Compound 5 in DMSO

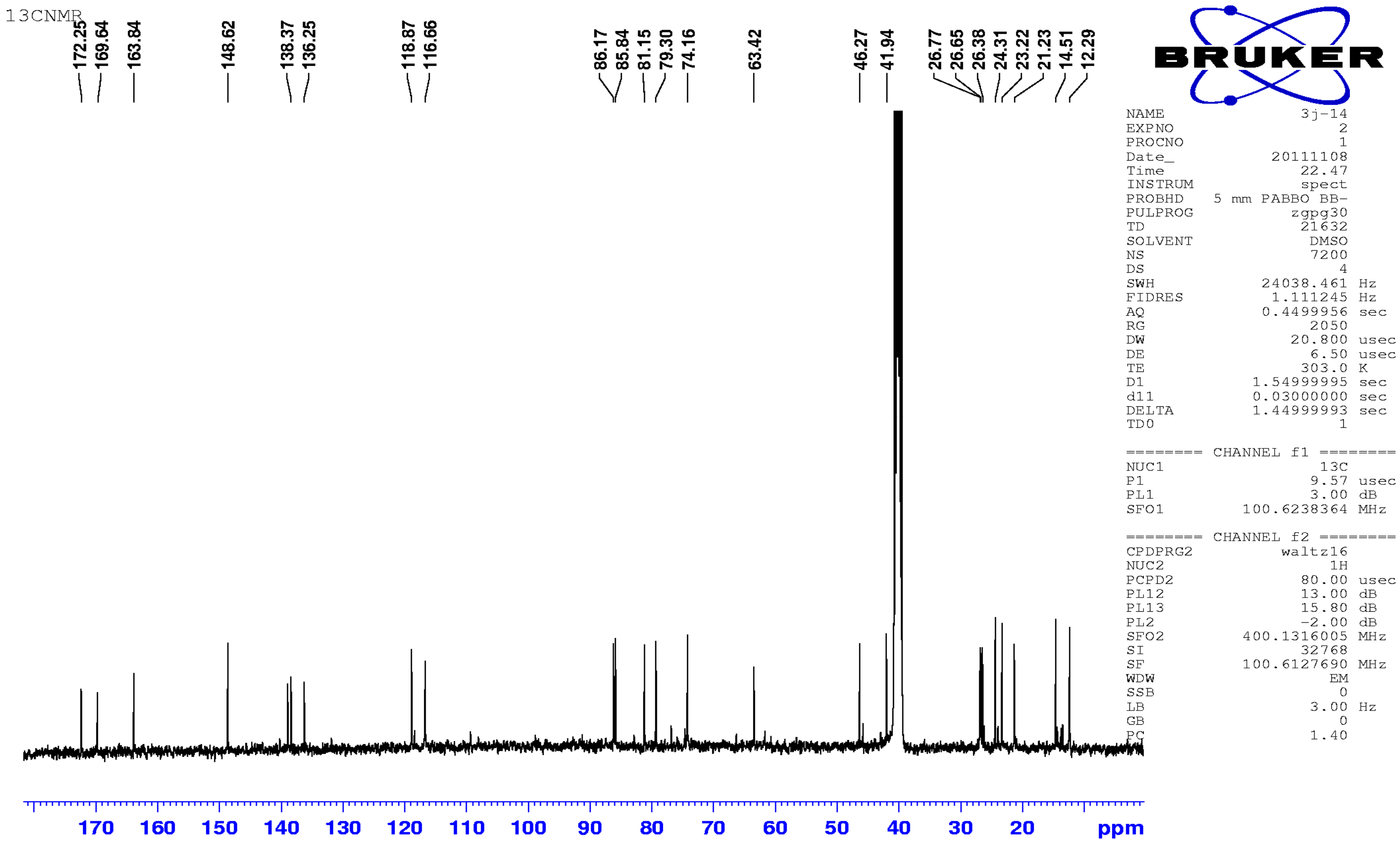

The ^{1}H NMR (400 MHz) spectrum of Compound 6 in DMSO

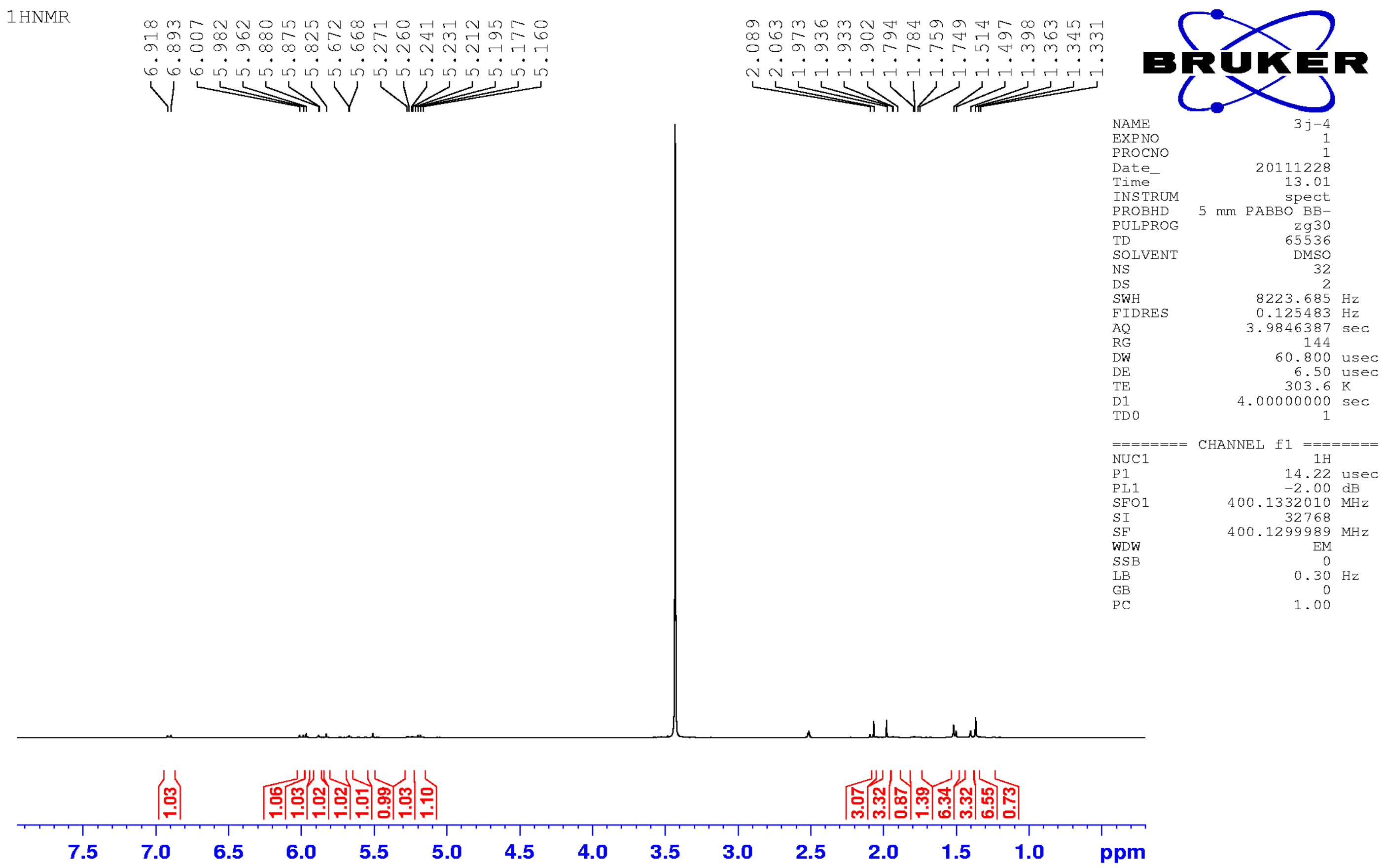

The ^{13}C NMR (400 MHz) spectrum of Compound 6 in DMSO

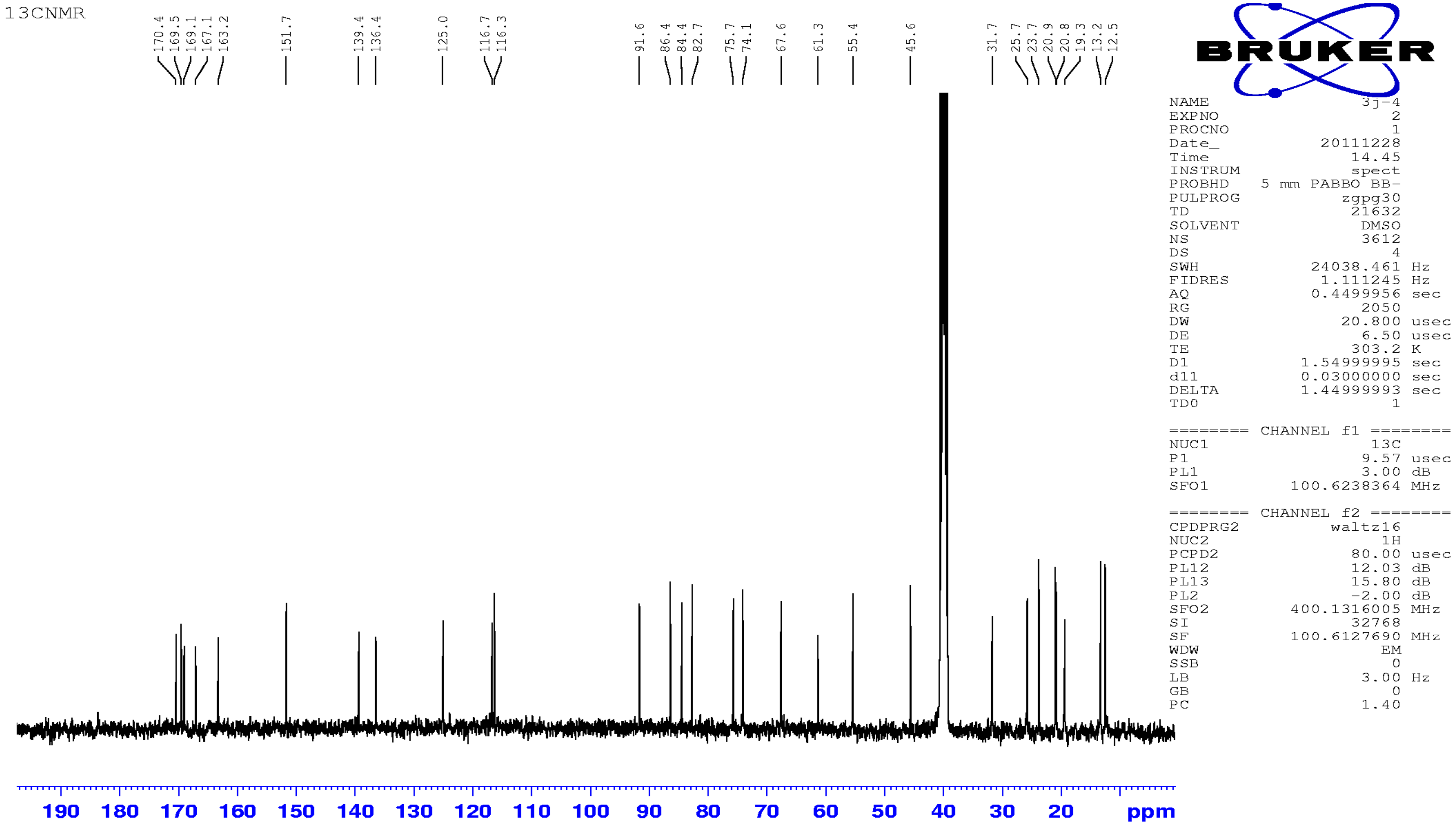

The ^{1}H-NMR (400 MHz) spectrum of Compound 7 in DMSO

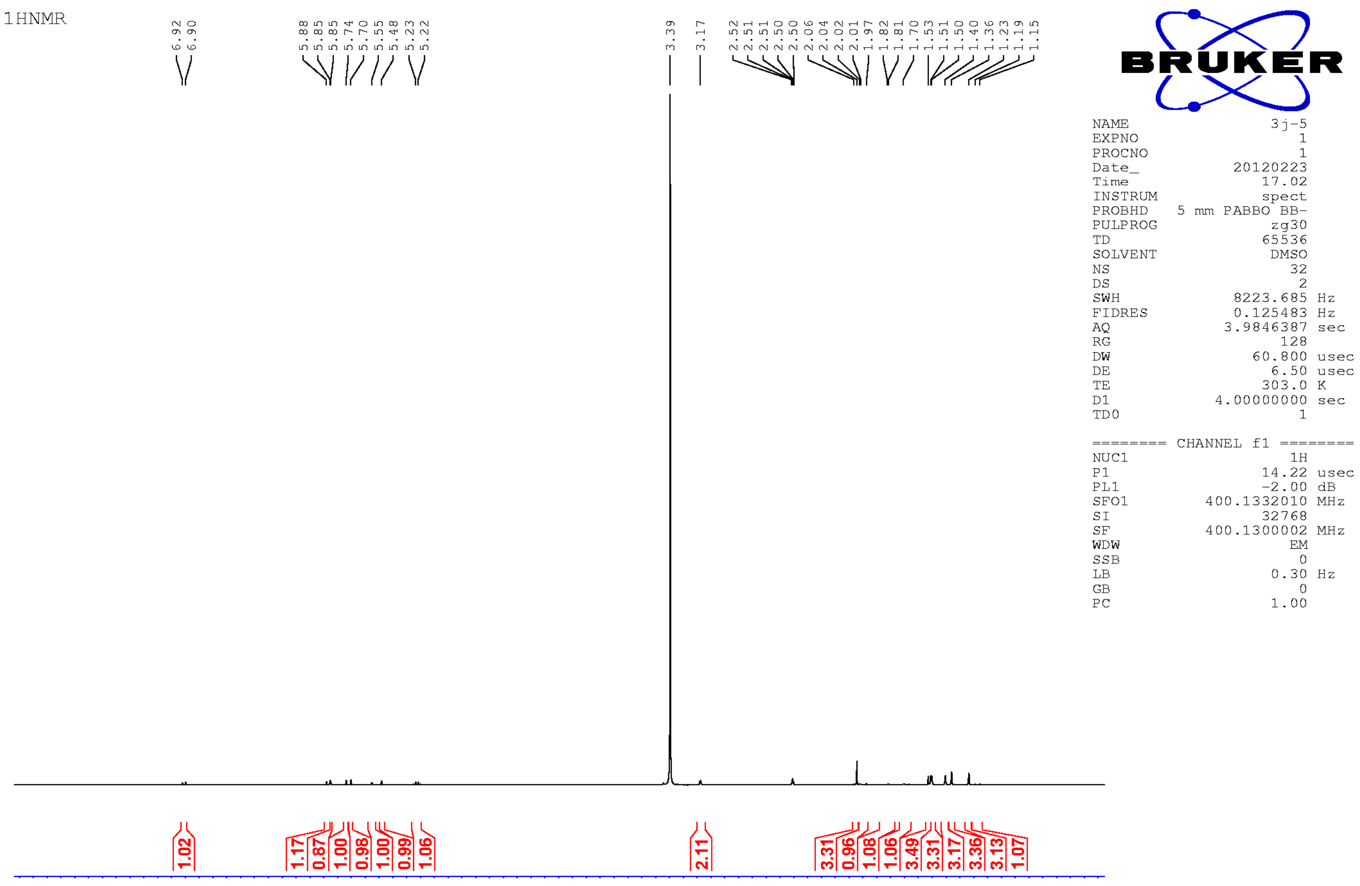

The ^{13}C-NMR (400 MHz) spectrum of Compound 7 in DMSO

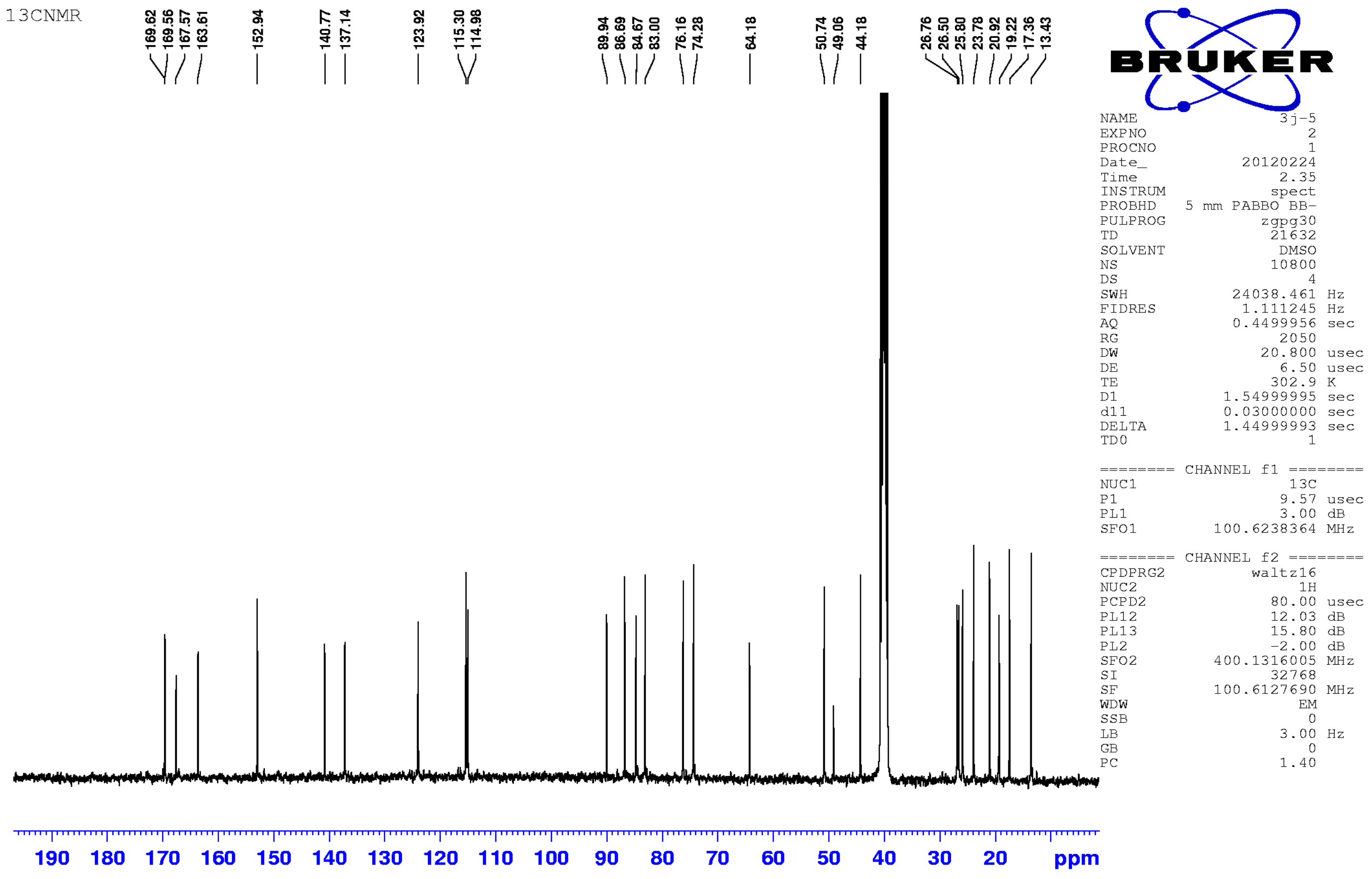

The ^{1}H-NMR (400 MHz) spectrum of Compound 8 in Acetone-d_6

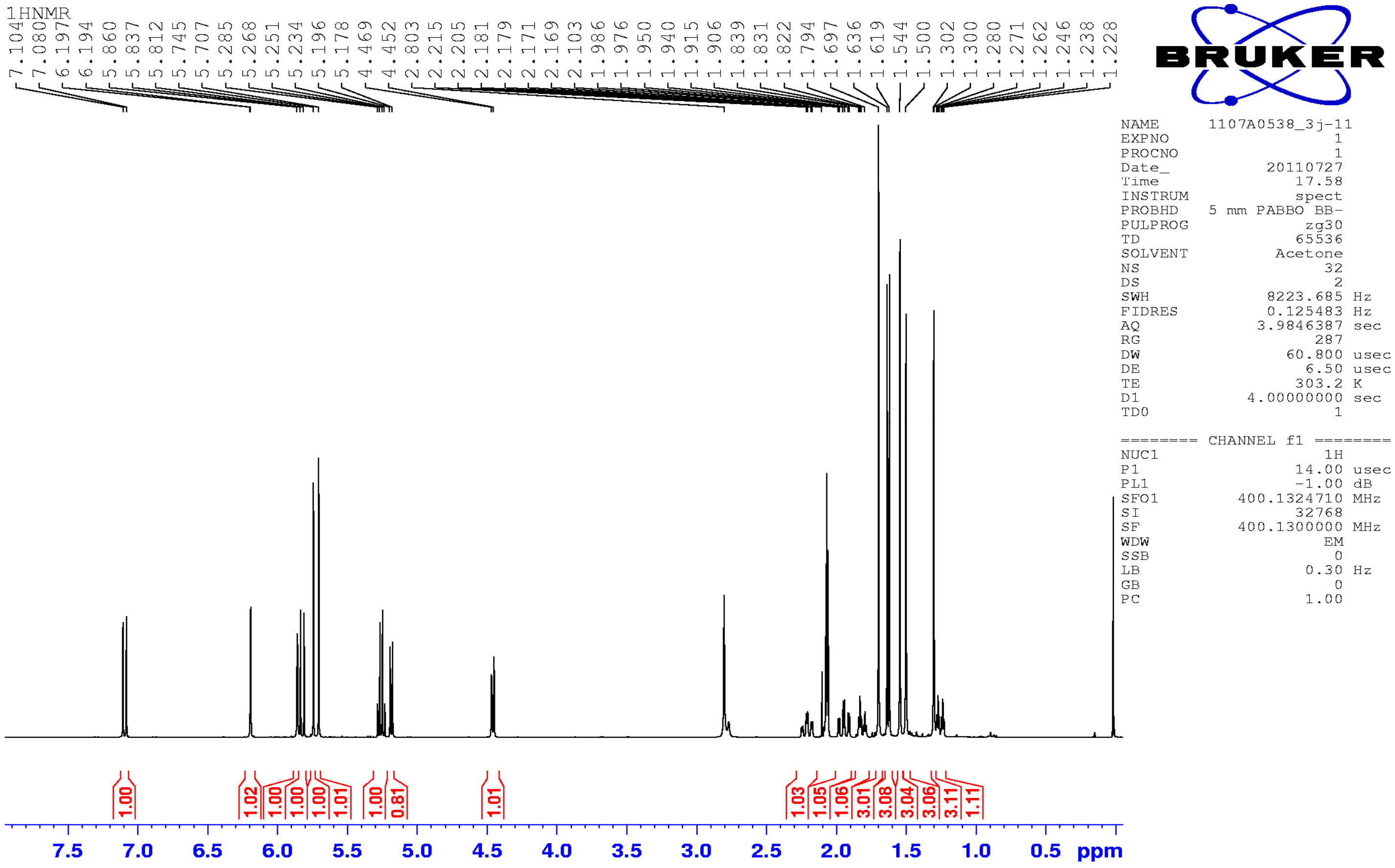

The ^{13}C-NMR (400 MHz) spectrum of Compound 8 in Acetone-d_6

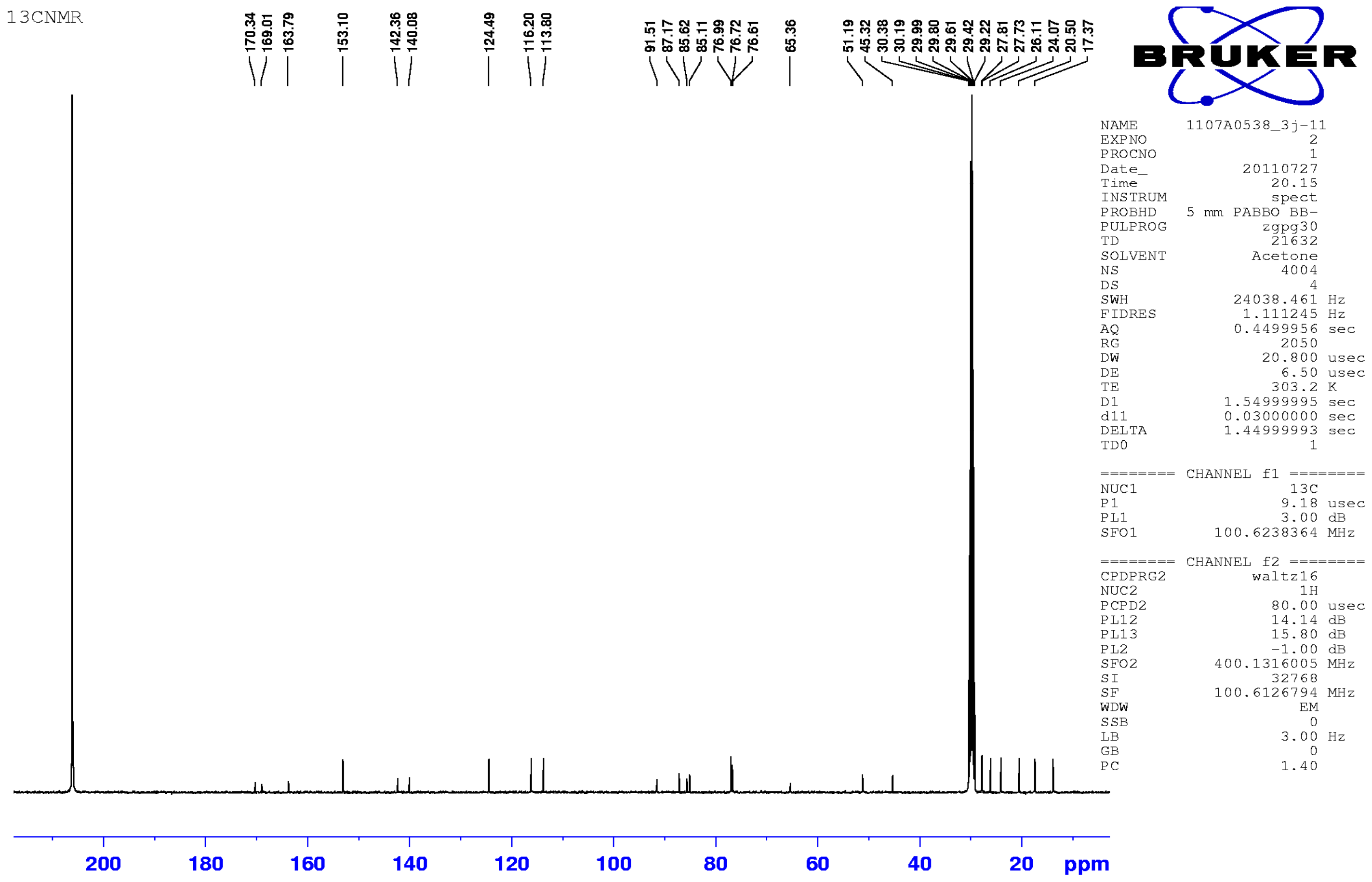

The ^{1}H-NMR (400 MHz) spectrum of Compound 9 in $CDCl_3$

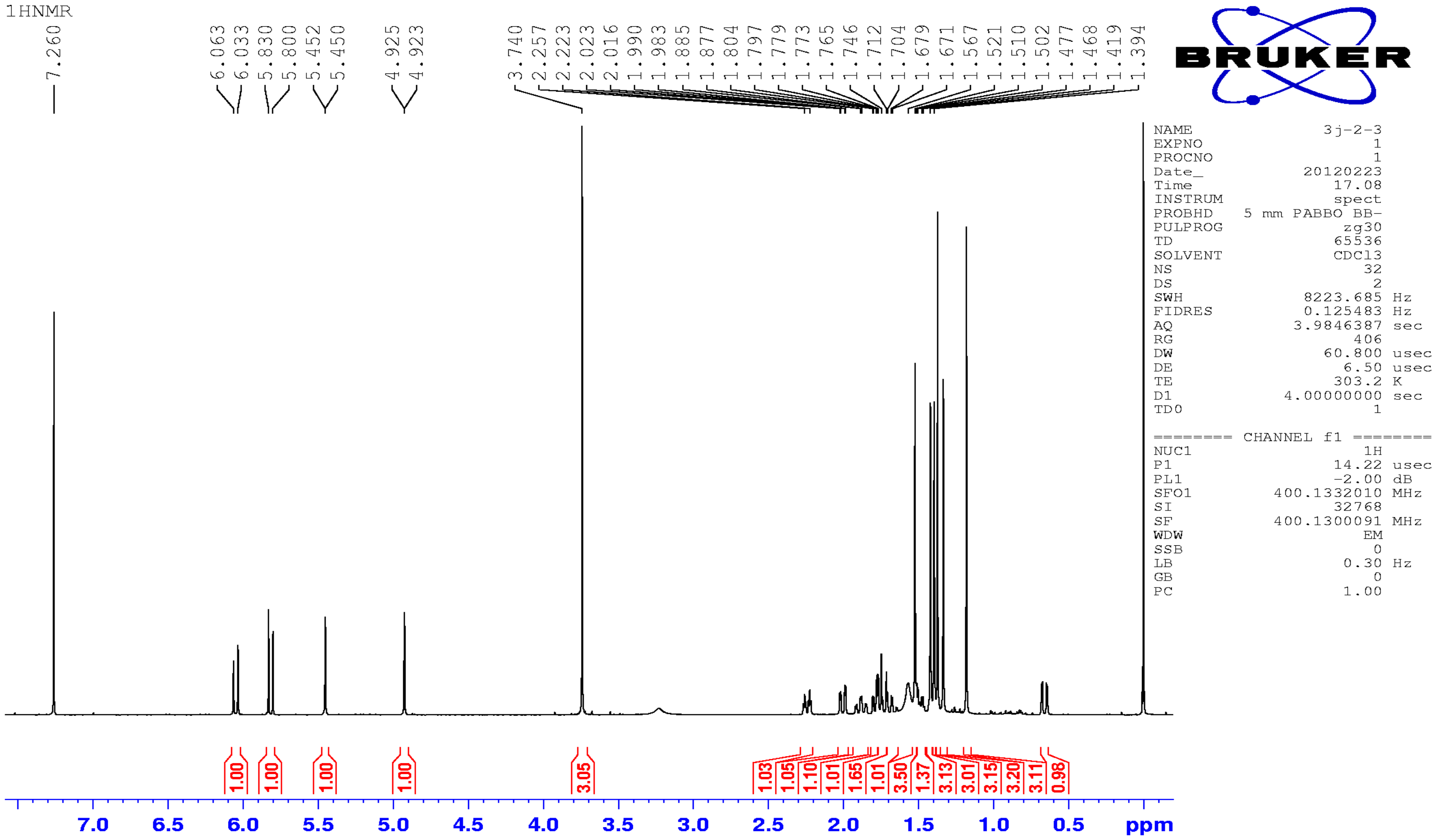

The ^{13}C-NMR (400 MHz) spectrum of Compound 9 in $CDCl_3$

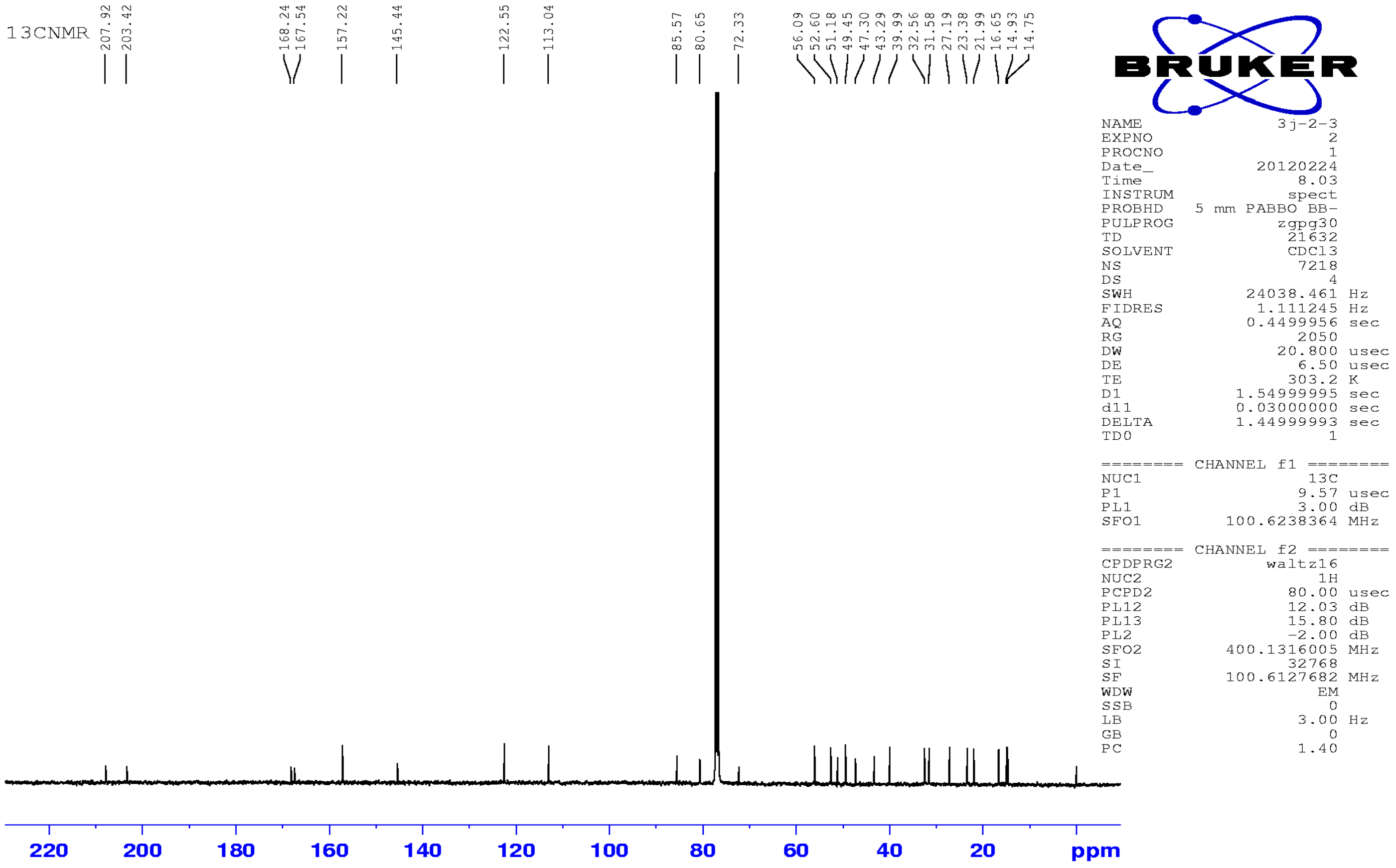

The ^{1}H-NMR (400 MHz) spectrum of Compound 10 in $CDCl_3$

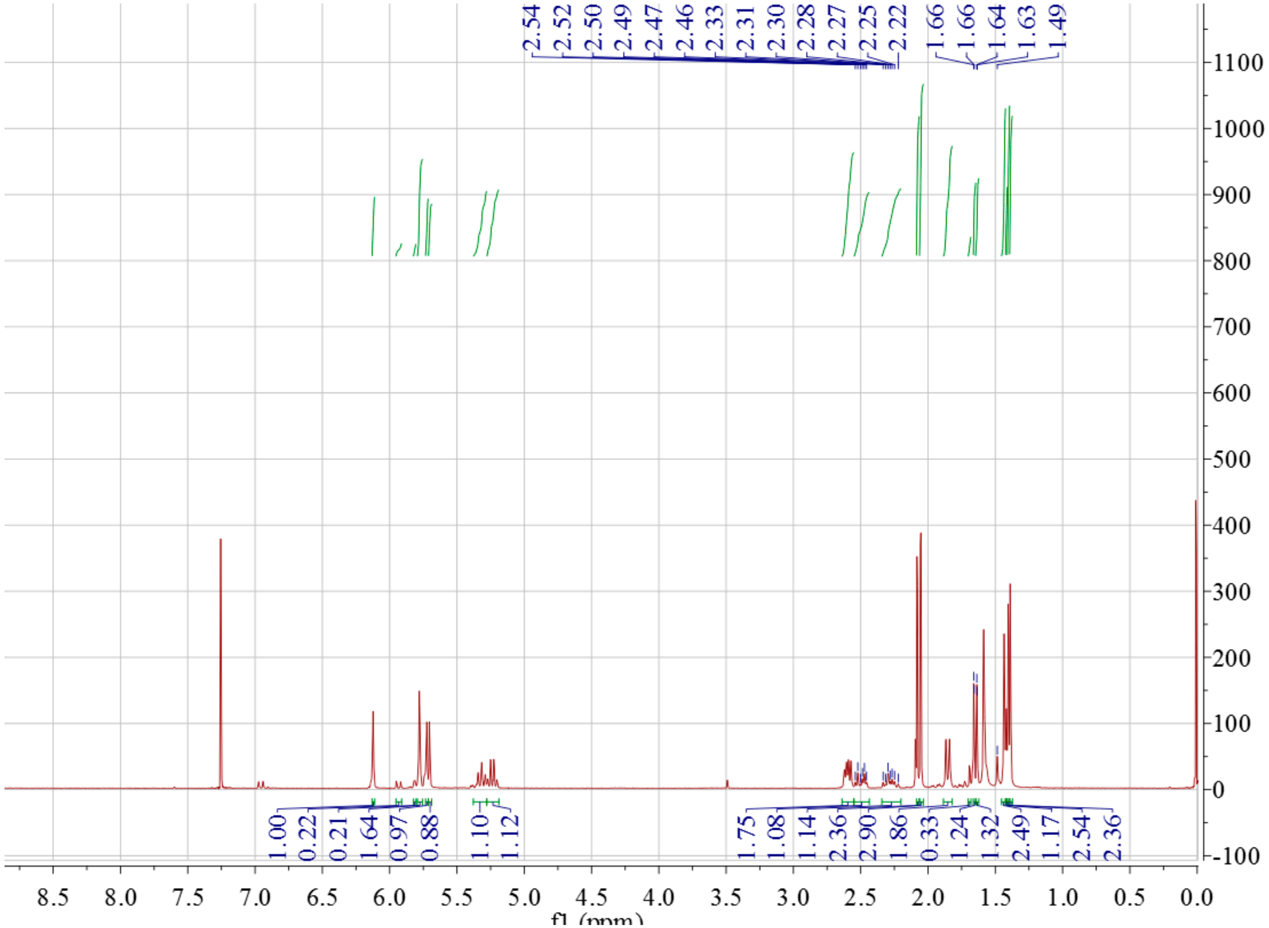

The ^{13}C-NMR (400 MHz) spectrum of Compound 10 in CDCl$_3$

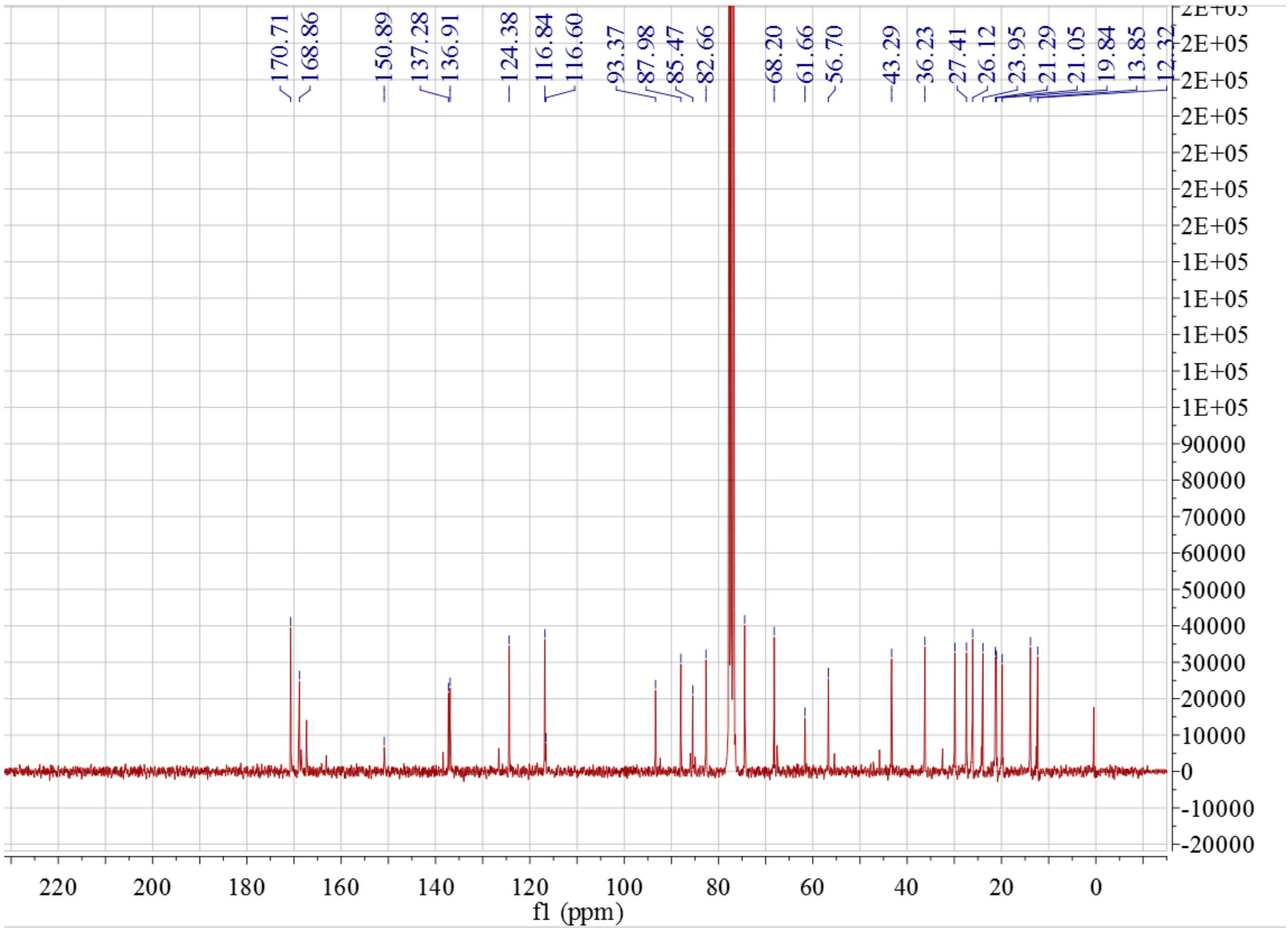

X-ray Crystallographic

Perspective ORTEP drawings for Compounds **1,3,7,8, 9**

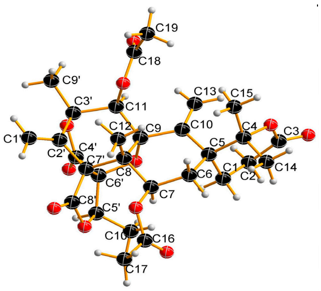

1

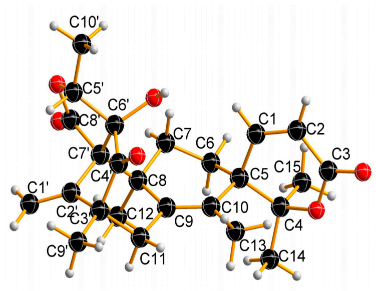

3

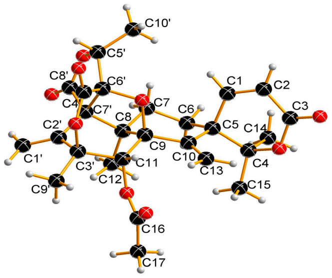

7

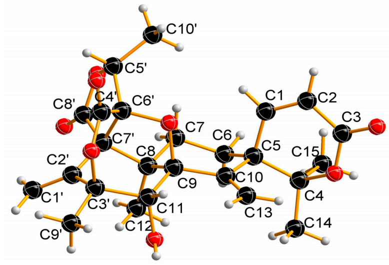

8

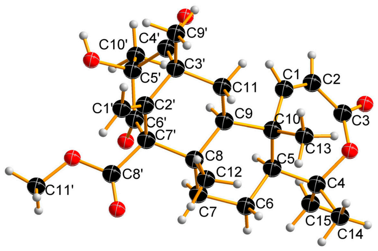

9

The ESIMS of Compound 1

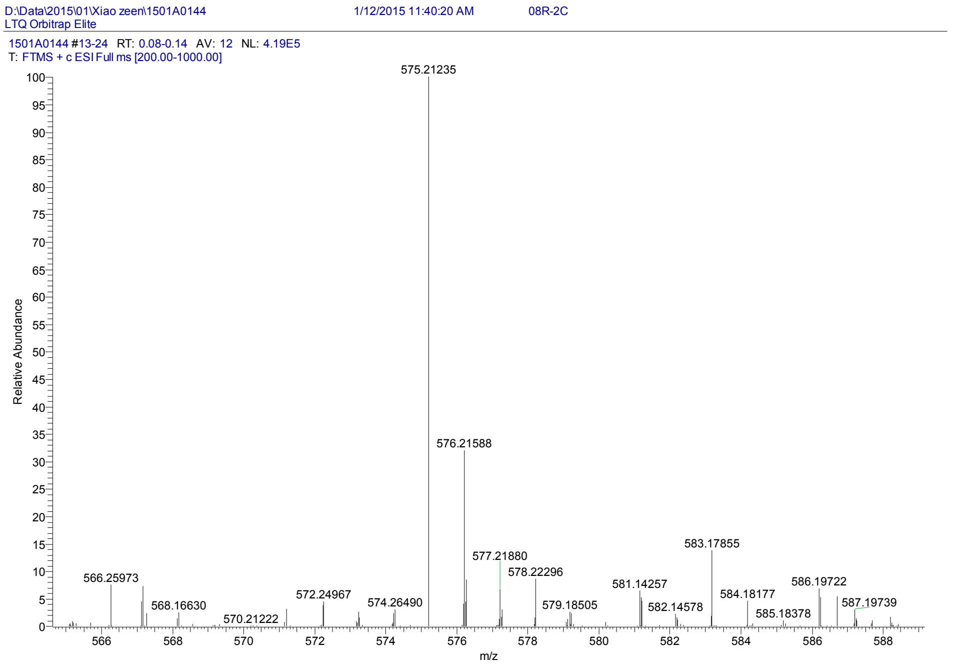

The HRESIMS of Compound 1

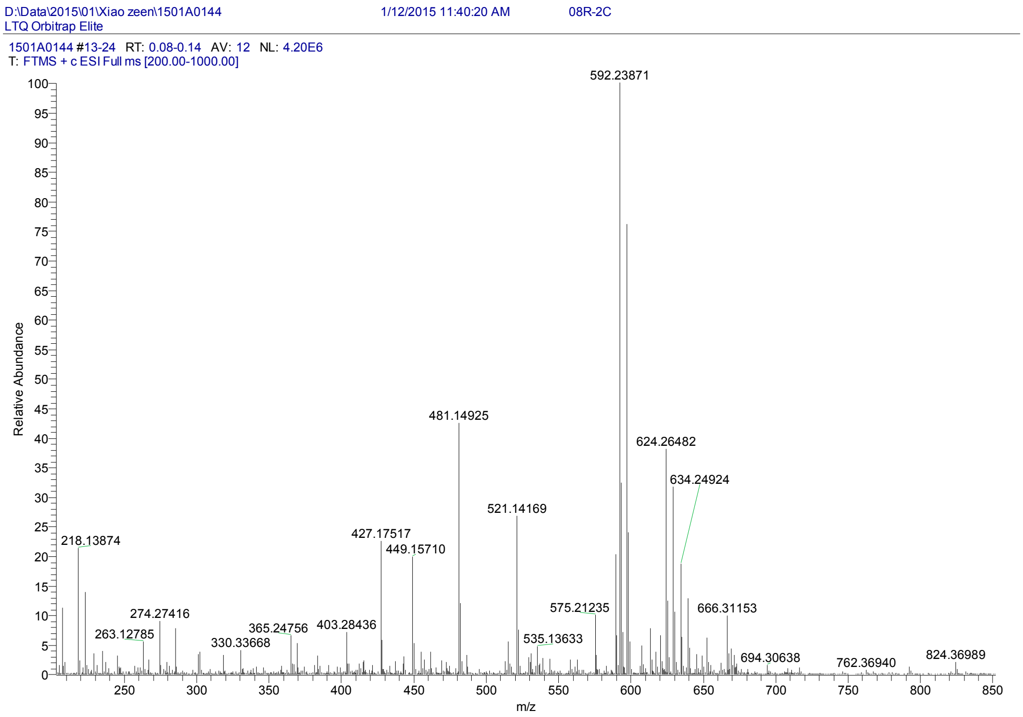

The ESIMS of Compound 2

Formula Predictor Report - 3j-3.lcd

Page 1 of 1

Data File: F:\data\wangjun\luyu\20120621\3j-3.lcd

Elmt	Val.	Min	Max	Elmt	Val.	Min	Max	Elmt	Val.	Min	Max	Elmt	Val.	Min	Max	Use Adduct
H	1	0	35	O	2	0	11	S	2	0	0	Pt	2	0	0	H
B	3	0	0	18O	2	0	0	Cl	1	0	5					Na
C	4	0	30	F	1	0	0	Br	1	0	5					
N	3	0	0	P	3	0	0	I	3	0	0					

Error Margin (ppm): 500
HC Ratio: unlimited
Max Isotopes: all
MSn Iso RI (%): 75.00

DBE Range: not fixed
Apply N Rule: yes
Isotope RI (%): 1.00
MSn Logic Mode: AND

Electron Ions: both
Use MSn Info: yes
Isotope Res: 10000
Max Results: 500

Event#: 1 MS(E+) Ret. Time : 0.207 -> 0.273 Scan# : 63 -> 83

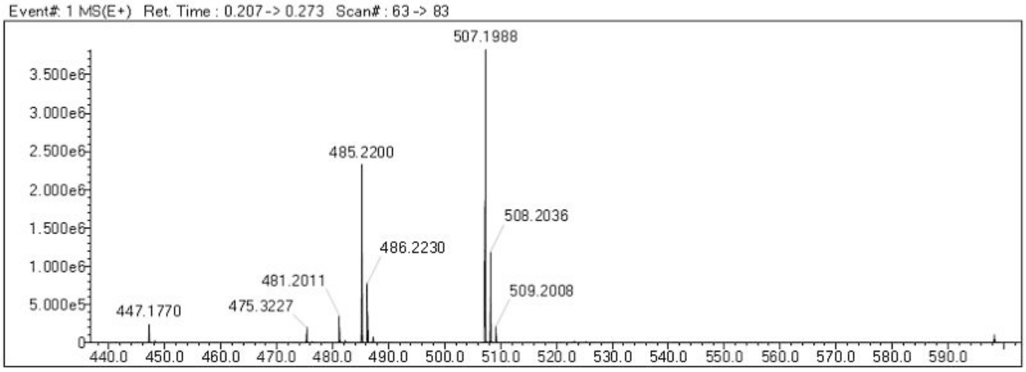

The HRESIMS of Compound 2

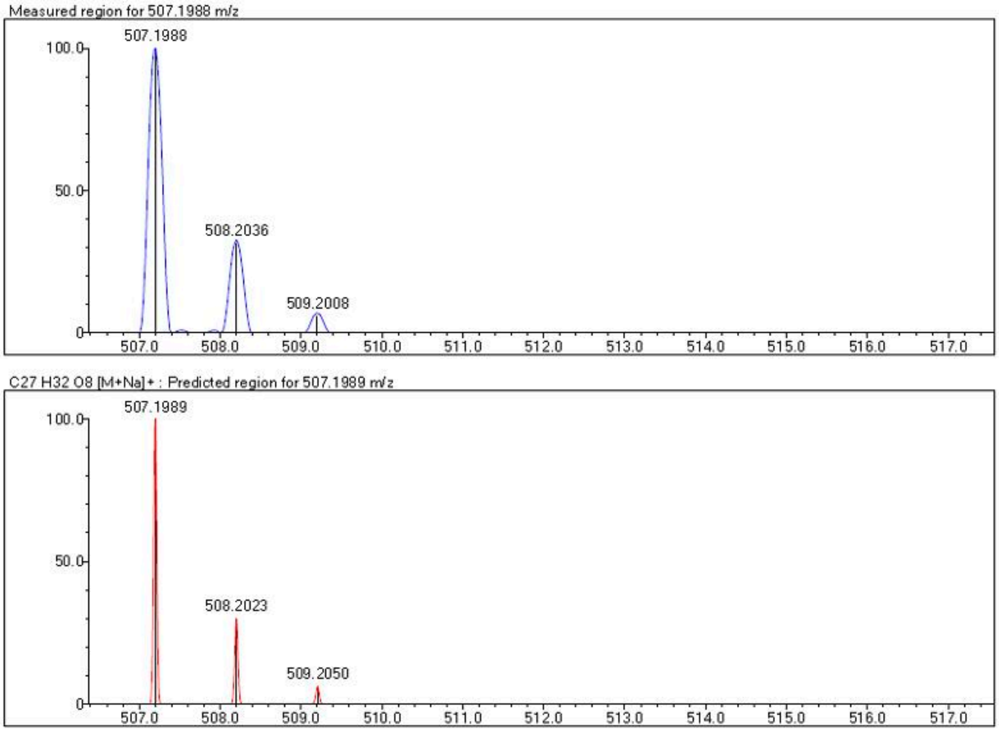

Rank	Score	Formula (M)	Ion	Meas. m/z	Pred. m/z	Df. (mDa)	Df. (ppm)	Iso	DBE
1	71.25	C27 H32 O8	[M+Na]+	507.1988	507.1989	-0.1	-0.20	71.25	12.0
